# Deep Learning Paradigm for Cardiovascular Disease/Stroke Risk Stratification in Parkinson’s Disease Affected by COVID-19: A Narrative Review

**DOI:** 10.3390/diagnostics12071543

**Published:** 2022-06-24

**Authors:** Jasjit S. Suri, Mahesh A. Maindarkar, Sudip Paul, Puneet Ahluwalia, Mrinalini Bhagawati, Luca Saba, Gavino Faa, Sanjay Saxena, Inder M. Singh, Paramjit S. Chadha, Monika Turk, Amer Johri, Narendra N. Khanna, Klaudija Viskovic, Sofia Mavrogeni, John R. Laird, Martin Miner, David W. Sobel, Antonella Balestrieri, Petros P. Sfikakis, George Tsoulfas, Athanase D. Protogerou, Durga Prasanna Misra, Vikas Agarwal, George D. Kitas, Raghu Kolluri, Jagjit S. Teji, Mustafa Al-Maini, Surinder K. Dhanjil, Meyypan Sockalingam, Ajit Saxena, Aditya Sharma, Vijay Rathore, Mostafa Fatemi, Azra Alizad, Padukode R. Krishnan, Tomaz Omerzu, Subbaram Naidu, Andrew Nicolaides, Kosmas I. Paraskevas, Mannudeep Kalra, Zoltán Ruzsa, Mostafa M. Fouda

**Affiliations:** 1 Stroke Monitoring and Diagnostic Division, AtheroPoint™, Roseville, CA 95661, USA; mahesh.nehu.333@gmail.com (M.A.M.); drindersingh1@gmail.com (I.M.S.); pomchadha@gmail.com (P.S.C.); surinderdhanjil@gmail.com (S.K.D.); 2 Department of Biomedical Engineering, North Eastern Hill University, Shillong 793022, India; sudip.paul.bhu@gmail.com (S.P.); bhagawatimrinalini07@gmail.com (M.B.); 3 Max Institute of Cancer Care, Max Super Specialty Hospital, New Delhi 110017, India; puneet1923@gmail.com; 4 Department of Radiology, and Pathology, Azienda Ospedaliero Universitaria, 09123 Cagliari, Italy; lucasabamd@gmail.com (L.S.); gavinofaa@gmail.com (G.F.); 5 Department of CSE, International Institute of Information Technology, Bhuneshwar 751029, India; sanjay@iiit-bh.ac.in; 6 Department of Neurology, University Medical Centre Maribor, 2000 Maribor, Slovenia; monika.turk84@gmail.com (M.T.); omerzu.tomaz@gmail.com (T.O.); 7 Department of Medicine, Division of Cardiology, Queen’s University, Kingston, ON K7L 3N6, Canada; amerschedule@gmail.com; 8 Department of Cardiology, Indraprastha APOLLO Hospitals, New Delhi 110076, India; drnnkhanna@gmail.com (N.N.K.); ajitsaxena@hotmail.com (A.S.); 9 Department of Radiology and Ultrasound, University Hospital for Infectious Diseases, 10000 Zagreb, Croatia; klaudija.viskovic@bfm.hr; 10 Cardiology Clinic, Onassis Cardiac Surgery Centre, 176 74 Athens, Greece; sophie.mavrogeni@gmail.com; 11 Heart and Vascular Institute, Adventist Health St. Helena, St. Helena, CA 94574, USA; lairdjr@ah.org; 12 Men’s Health Centre, Miriam Hospital, Providence, RI 02906, USA; martin_miner@brown.edu; 13 Rheumatology Unit, National Kapodistrian University of Athens, 157 72 Athens, Greece; dwsobel@gmail.com (D.W.S.); psfikakis@med.uoa.gr (P.P.S.); 14 Docs Eye Care Research Lab, Dunedin 9013, New Zealand; antonellabalestrieri@hotmail.com; 15 Department of Surgery, Aristoteleion University of Thessaloniki, 541 24 Thessaloniki, Greece; tsoulfasg@gmail.com; 16 Cardiovascular Prevention and Research Unit, Department of Pathophysiology, National & Kapodistrian University of Athens, 157 72 Athens, Greece; aprotog@med.uoa.gr; 17 Sanjay Gandhi Postgraduate Institute of Medical Sciences, Lucknow 226014, India; durgapmisra@gmail.com (D.P.M.); vikasagr@yahoo.com (V.A.); 18 Academic Affairs, Dudley Group NHS Foundation Trust, Dudley DY1 2HQ, UK; george.kitas@nhs.net; 19 Arthritis Research UK Epidemiology Unit, Manchester University, Manchester M13 9PL, UK; 20 OhioHealth Heart and Vascular, Mansfield, OH 44905, USA; kolluri.raghu@gmail.com; 21 Ann and Robert H. Lurie Children’s Hospital of Chicago, Chicago, IL 60611, USA; jteji@mercy-chicago.org; 22 Allergy, Clinical Immunology, and Rheumatology Institute, Toronto, ON M5G 1N8, Canada; almaini@hotmail.com; 23 MV Centre of Diabetes, Chennai 600013, India; dr_chokku@yahoo.com; 24 Division of Cardiovascular Medicine, University of Virginia, Charlottesville, VA 22908, USA; as8ah@hscmail.mcc.virginia.edu; 25 Nephrology Department, Kaiser Permanente, Sacramento, CA 95823, USA; vijay.s.rathore@kp.org; 26 Department of Physiology & Biomedical Engineering, Mayo Clinic College of Medicine and Science, Rochester, MN 55905, USA; fatemi.mostafa@mayo.edu; 27 Department of Radiology, Mayo Clinic College of Medicine and Science, Rochester, MN 55905, USA; azra.alizad@gmail.com; 28 Neurology Department, Fortis Hospital, Bangalore 560076, India; pudukode.krishnan@fortisheakthcare.com; 29 Electrical Engineering Department, University of Minnesota, Duluth, MN 55812, USA; dsnaidu@d.umn.edu; 30 Vascular Screening and Diagnostic Centre, University of Nicosia Medical School, Engomi 2408, Cyprus; anicolaides1@gmail.com; 31 Department of Vascular Surgery, Central Clinic of Athens, 106 80 Athens, Greece; paraskevask@hotmail.com; 32 Department of Radiology, Harvard Medical School, Boston, MA 02115, USA; mkalra@mgh.harvard.edu; 33 Invasive Cardiology Division, Faculty of Medicine, University of Szeged, 6720 Szeged, Hungary; zruzsa25@gmail.com; 34 Department of Electrical and Computer Engineering, Idaho State University, Pocatello, ID 83209, USA; mfouda@ieee.org

**Keywords:** Parkinson’s disease, COVID-19, cardiovascular/stroke risk stratification, deep learning, bias

## Abstract

***Background and Motivation***: Parkinson’s disease (PD) is one of the most serious, non-curable, and expensive to treat. Recently, machine learning (ML) has shown to be able to predict cardiovascular/stroke risk in PD patients. The presence of COVID-19 causes the ML systems to become severely non-linear and poses challenges in cardiovascular/stroke risk stratification. Further, due to comorbidity, sample size constraints, and poor scientific and clinical validation techniques, there have been no well-explained ML paradigms. Deep neural networks are powerful learning machines that generalize non-linear conditions. This study presents a novel investigation of deep learning (DL) solutions for CVD/stroke risk prediction in PD patients affected by the COVID-19 framework. ***Method***: The PRISMA search strategy was used for the selection of 292 studies closely associated with the effect of PD on CVD risk in the COVID-19 framework. We study the hypothesis that PD in the presence of COVID-19 can cause more harm to the heart and brain than in non-COVID-19 conditions. COVID-19 lung damage severity can be used as a covariate during DL training model designs. We, therefore, propose a DL model for the estimation of, (i) COVID-19 lesions in computed tomography (CT) scans and (ii) combining the covariates of PD, COVID-19 lesions, office and laboratory arterial atherosclerotic image-based biomarkers, and medicine usage for the PD patients for the design of DL point-based models for CVD/stroke risk stratification. ***Results***: We validated the feasibility of CVD/stroke risk stratification in PD patients in the presence of a COVID-19 environment and this was also verified. DL architectures like long short-term memory (LSTM), and recurrent neural network (RNN) were studied for CVD/stroke risk stratification showing powerful designs. Lastly, we examined the artificial intelligence bias and provided recommendations for early detection of CVD/stroke in PD patients in the presence of COVID-19. ***Conclusion***: The DL is a very powerful tool for predicting CVD/stroke risk in PD patients affected by COVID-19.

## 1. Introduction

Parkinson’s disease (PD) is a progressive neurodegenerative condition characterized by movement impairments. In 1817, British physician James Parkinson described the condition for the first time [1]. PD is characterized by the loss and dysfunction of neurons (nerve cells) in the substantia nigra, a region of the brain. PD is characterized by problems with dopamine pathways, which are cells in the brain that communicate with other neurons by creating dopamine, also called a neurotransmitter [2]. According to data from a variety of studies, the cost of treating and controlling PD is expensive [3]. Western nations have more PD cases than Asian countries. [3,4].

A coronavirus 2 (SARS-CoV-2)-related acute, respiratory distress disease was found in Wuhan, China, in late December 2019 [5,6]. The infection spread fast over the world, resulting in a global coronavirus pandemic in 2020. Between 31 December 2019, and 11 March 2022, there were nearly 450,229,635 cases of COVID-19 reported globally, with around 6,019,085 deaths [7]. PD is found in the elder age group and it has been observed that PD patients show comorbidities such as diabetes [8], hypertension [9,10], dementia [11,12], chronic kidney, temporomandibular disorder (TMD) [13], and heart problems [14,15], and thus need continuous medical treatment to control the PD. However, due to the coronavirus pandemic, most countries declared a lockdown, and all the medical forces were used to control the spread of the COVID-19.

During the period of lockdown, less importance was given to PD-affected patients, leading to heart attack and stroke in PD patients [16]. The connection between cardiovascular disease (CVD), stroke, and PD with COVID-19 seems particularly important due to several observations. Antibodies against coronavirus were discovered in the cerebrospinal fluid in PD patients more than two decades ago, implying that viral infections may play a role in the neurodegenerative process [11,17].

As per recent studies [18,19,20], PD increases the risk of heart attack and stroke [21,22]; current study indicates that PD is associated with vascular risk factors such as diabetes and hypertension [23,24,25,26,27]. Thus, CVD/stroke risk early detection becomes even more important during the joint effects of PD with COVID-19, as it increases the risk of mortality [28]. However, during the joint effects, the covariates cause non-linearity between the covariates and the gold standard. Thus, special tools are needed for CVD/stroke risk stratification [4]. Since COVID-19 causes a change in the risk factors in PD patients, we need a self-adjusting system that can automatically estimate the risk of CVD/stroke in PD patients when COVID-19 is triggered [29].

In recent years, it has been seen that artificial intelligence (AI) has played an important role in computer-aided diagnosis [30,31], particularly in the identification and classification of multiple diseases [32,33,34,35]. The application of machine learning (ML) has recently been explained to have dominated the field of medical imaging, including diabetes [36,37], cardiovascular disease [38], liver [33], cancers such as thyroid [39,40], vascular screening [41], ovarian [42], prostate [43], risk characterization using coronary and vascular screening [41], and carotid angiography [44]. Many medical imaging modalities are available for imaging, including magnetic resonance imaging (MRI) [45,46], computed tomography (CT) [47], ultrasonography (US) [48], and CT for lung imaging, all of which can illustrate COVID-19 symptoms and lesions [45,46]. It has been shown that the DL algorithm can segment COVID-19 lungs and has been utilized to detect the lesions in CT lung images on four separate occasions [45,49,50]. Since the PD dataset comprises a variety of motor symptoms, variables that have been observed in previous studies, AI models have also been developed to predict the disease [51,52,53]. Therefore, we believe that DL systems will be useful in the future for forecasting CVD/stroke risk classification in PD patients within the COVID-19 framework, and that developing a design approach will be beneficial in the near future.

The focus of this research was to design, develop, and validate the hypothesis, (i) CVD/stroke risk stratification of PD patients in the COVID-19 framework and (ii) understanding the non-linearity of PD combined with COVID-19 symptoms against the CVD/stroke gold standard; (iii) develop a DL-based lesion detection system and its quantification, which could then be used as a covariate in a machine learning framework; (iv) DL-based CVD/stroke risk stratification by combining office-based biomarkers (OBBMs), laboratory-based biomarkers (LBBMs), carotid ultrasound image-based (CUSIP), medicine usage (MedUSE), PD-based biomarkers, and CT-based COVID-19 lesion biomarkers.

## 2. Search Strategy

IEEE Xplore, Google Scholar, PubMed, and Science Direct were used to conduct an overall writing search. ‘CHD’, ‘PD patients and stroke risk stratification, ‘neurodegenerative disease and symptoms’, ‘AI’, ‘machine learning, ‘deep learning, and ‘neurodegenerative disease’ were some of the key watchwords used in the study selection process. The research article was chosen for the studies that cover a wide range of topics, including CVD, and stroke risk stratification of PD patients in the COVID-19 framework using ML, DL, hybrid deep learning (HDL), and AI. The categorization of normal vs. PD-affected persons.

**Figure 1:**
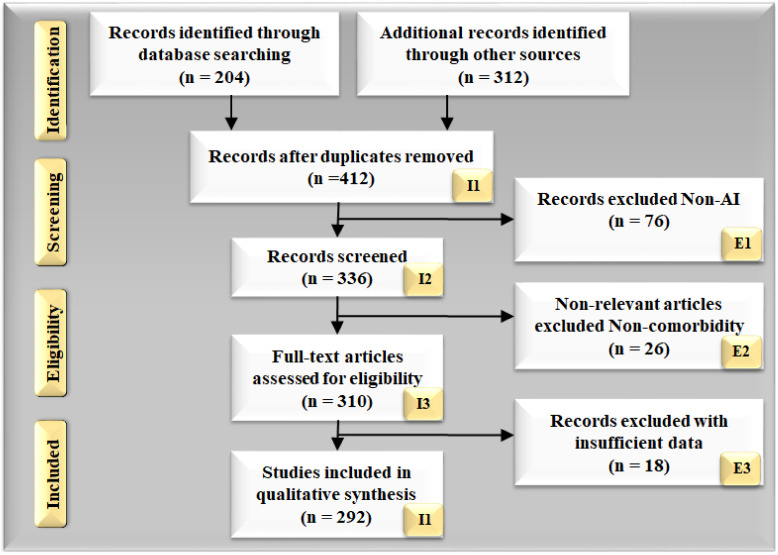
PRISMA model for selection of the studies, dealing with the effect of COVID-19 on PD for CVD and stroke risk stratification. (I: Included, E: Excluded).

Figure 1 demonstrates the PRISMA model for the research article selection approach. Almost 204 research articles were identified from the listed sources, and 312 research studies were identified from additional sources during the identification phase. Articles that cross the research aim or have duplications were deleted from the total 412 studies. Articles were evaluated based on the viability of the selection strategy’s goal (336 studies). The papers that were not AI-based (n = 76) were ignored. Many of the articles did not meet domain requirements for reasons such as insufficient data, information, or poor presentation. As a result, the analysis was based on a total of 292 studies.

Information from the data was considered for the PD with COVID-19 studies data, searches were: (i) name of the author, (ii) publication year of research article, (iii) objective of the research studies, (iv) effect of COVID-19 on PD, heart, and brain, (v) PD year (vi), PD with other comorbidities, (vii) diagnosis method, (viii) PD symptoms worsening factor due to COVID-19, and (ix) treatment of PD with COVID-19. The identified research studies were assessed using the unique and effective application of the AI, hybrid AI, PD with other comorbidities diagnosis techniques, and biomarker-based strategies for detecting CVD and stroke risk stratification of PD patients in the COVID-19 framework.

**Figure 2:**
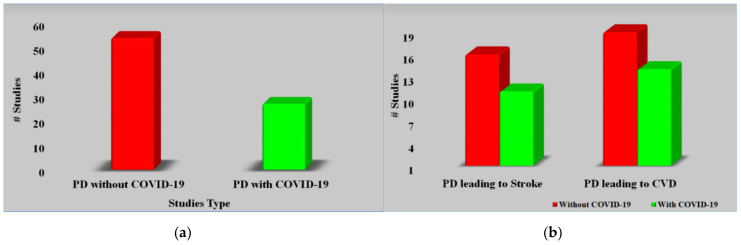
(**a**) Studies related to PD with or without COVID-19. (**b**) Studies related to PD leading to stroke and CVD with or without COVID-19.

Figure 2a, studies related to PD with or without COVID-19. Figure 2b represents studies related to PD leading to stroke and heart disease with or without COVID-19. Every study was examined using a feasibility analysis and then cross-checked using scientific validation to ensure that it closely matched our objectives.

## 3. Pathophysiology of Lung and Parkinson’s Disease during COVID-19

The effect of COVID-19 on PD, the heart, and the brain is still unknown due to the limited literature, especially since we know that the lungs are badly affected by COVID-19 [54,55]. Note that PD patients already have several other comorbidities due to the older age group [6,16,56]. The symptoms related to PD with COVID-19 and other comorbidities introduce nonlinearity, causing challenges in the ML system for CVD/stroke risk stratification [16].

### 3.1. Acute Respiratory Distress Syndrome, Imaging, and Lung Lesions during COVID-19

**Figure 3:**
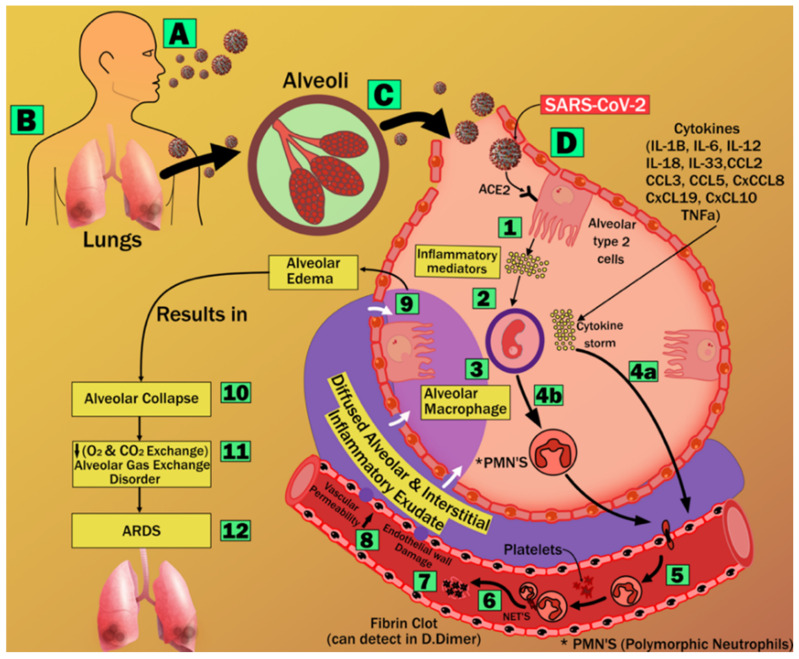
Stages of acute respiratory distress syndrome formation [69].

The effect of coronavirus on lungs results in lower levels of ACE2 proliferating in the lung parenchyma cells. It leads to exacerbated neutrophil buildup, increased vascular permeability, and the production of diffuse alveolar and interstitial exudates in the lung. Pneumonia and acute respiratory distress syndrome (ARDS) are the results of this process [57]. As a result of an oxygen and carbon dioxide imbalance, ARDS is characterized by significant anomalies in blood gas composition that result in low blood oxygen levels [58]. This chronic hypoxia has been shown to cause myocardial ischemia and cardiac damage [59,60]. In the brain, hypoxia increases the rate of anaerobic metabolism in the mitochondrial brain cells [61], which results in increased cerebral vasodilation, edema, and decreased blood flow. There is a risk of cerebral ischemia and the development of acute cerebrovascular disorders, including acute ischemic stroke [61]. Figure 3 explains the pathway of ARDS formation.

To diagnose the abnormalities in the lung we need an imaging technique, and x-rays and computer tomography are the two medical imaging techniques that are most important in the detection and diagnosis of COVID-19 [47,62]. CT has demonstrated high sensitivity and repeatability. It also can detect various types of opacities, such as ground-glass opacity (GGO), consolidation, and other opacities [63,64], that are primarily seen [65,66]. The potential of ML systems to mimic traditionally established processes is outstanding, and this allows for faster illness identification and diagnosis [64]. The most significant flaw in such models is the features extracting method, which is arbitrary and, as a result, time-consuming [64]. It has recently been demonstrated that DL models can overcome this challenge [67,68]. In AI, deep learning is a branch that makes use of deep layers to provide self-driving feature extraction, classification, and segmentation of data input. The details on DL-based lesion segmentation and quantification will be explained in Section 5.

### 3.2. Vascular Damage Due to COVID-19

**Figure 4:**
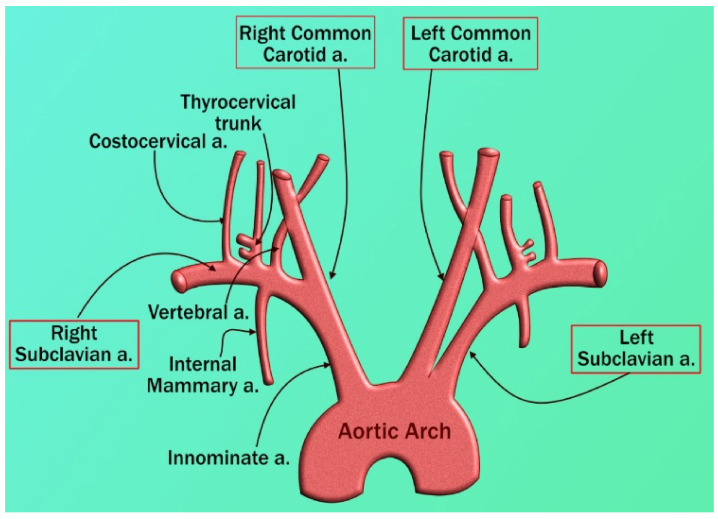
The inception of the left and right carotid arteries [69].

Vascular disorders create a threat to the heart and the brain [70]. The aortic arch, coronary artery, and carotid artery all have similar structures, this connection is extensively observed because the genetic composition of carotid and coronary arteries is similar (A to D) [71,72]. Even though they originate from a distinct main artery, these arteries run in opposite directions (Figure 4). Inflammatory cells such as polymorphonuclear cells, T-lymphocytes, histiocytes, monocytes, and mononuclear giant cells were found in all samples in the thrombus formation and all layers of vessels, along with endothelial proliferation and vascular endothelial, as well as collagen deposition and myofibroblastic proliferation to varying degrees. Endothelial damage can induce thrombosis in the arteries of the limbs and the aorta, as well as significant vascular events such as acute arterial hypoxia [73]. These cause LDL deposition and oxidation, plaque development, and arterial lumen constriction [74,75]. As a result, carotid artery disease may be used as a substitute biomarker for coronary artery disease in PD patients affected by COVID-19 [76]. COVID-19 is the cause of thrombosis in the arteries and veins and is also responsible for the unbalanced inflammatory state (cytokine storm) that also includes endothelial cells (1 to 12) [77].

### 3.3. Dopamine in Parkinson’s Disease with or without COVID-19

Dopamine acts as an intermediary between both the brain and the sensory systems that control and regulate movement [20]. The substantia nigra region of the brain loses nerve cells, causing PD. This part of the brain is responsible for producing dopamine, which is created by nerve cells [78]. This is a catalyst in the neuron neurodegeneration process because of the COVID-19 virus. Dopamine levels in the brain drop when these neuron cells die or are injured [79]. This shows that the brain area that governs movement is malfunctioning, resulting in delayed, unwanted, and uneven movements [80]. Nerve cell death is indeed a slow process. PD symptoms appear when roughly 80% of the substantia nigra cells in the brain are damaged [39,60].

**Figure 5:**
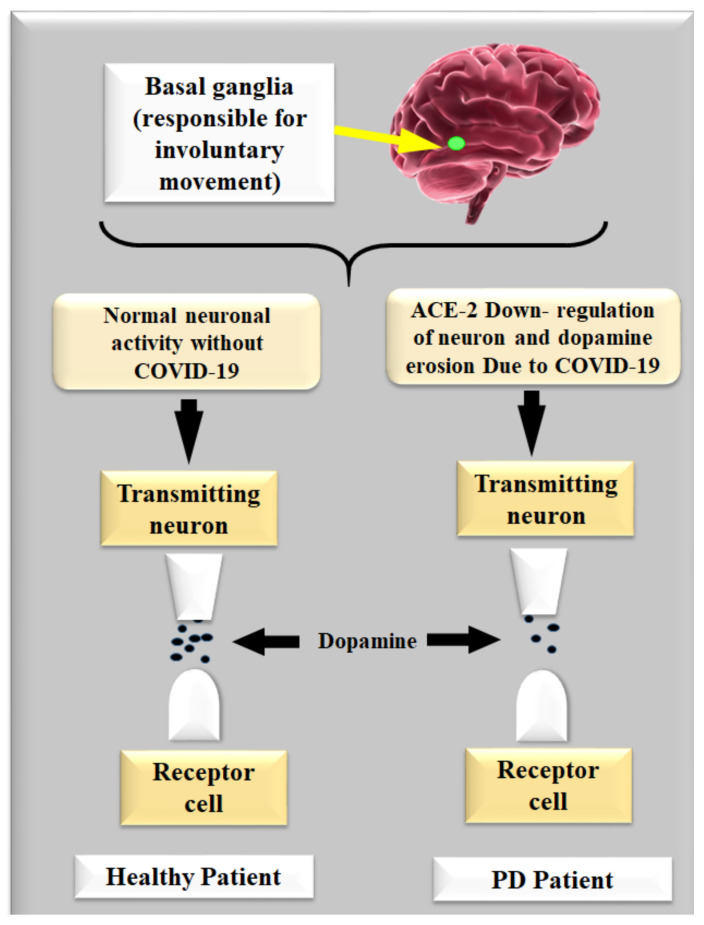
Effect of loss of dopamine in PD with or without COVID-19 (Courtesy of AtheroPoint, Roseville, CA, USA).

Figure 5 represents neurons leading to dopamine gradually reducing the basal ganglia, resulting in motor and non-motor abnormalities, such as, (i) motor system rigidity [1], bradykinesia [52,81], postural instability [82], faced mask, and hypophonic speech [52], and (ii) non-motor symptoms including constipation [83], autonomic dysfunction [83], dementia [84], depression and sleep disorder [83], behavioral problems, as well as issues adapting to changes and stresses in their environment [1].

The cause of developing PD is still unknown, there is no specific reason why it happens in the aging population. Environmental factors may potentially increase a person’s likelihood of developing PD [1]. However, it is still unclear how specific inherited and hereditary factors contribute to a person’s risk of developing PD [82,83]. The defective genes are passed down from one generation to the next, and PD can run in families [85]. There have been indications that pesticides and herbicides used in agriculture, as well as industrial pollutants and traffic, could also be contributing factors to PD [86].

## 4. The Relationship between Parkinson’s Disease, Heart, Brain, and COVID-19

The most common consequences of PD on the heart are heart failure, abrupt death, and edema [87]. As a result, PD is associated with an increased risk of dementia and a higher than average rate of mortality and morbidity [19,88]. Patients with PD commonly experience the symptoms and signs of tremor, bradykinesia, rigidity, and uncontrollable movements [89,90,91]. All of the pathological variables that contribute to the development of clinical complexity in PD include inappropriate protein aggregation, oxidative stress, neuroinflammation, mitochondrial damage, and genetic anomalies [92,93].

### 4.1. The Relationship between Parkinson’s Disease and CVD

It was shown that in 20–30% of hospitalized patients, 40% of COVID-19-related fatalities were due to cardiac damage [94]. Due to PD, it was observed that there was severe cardiac damage in ICU patients and it was 13 times higher than in non-ICU patients [95]. Further, it was noticed that due to PD, there was acute cardiac damage in 17%, death in 59%, and only 1% survived out of 191 individuals studied [96]. It was also noted that due to PD, the mortality risk associated with acute cardiac damage was shown to be far greater than the risk associated with age, diabetes, chronic obstructive pulmonary disease (COPD), or previous CVD history [38].

An artery’s epithelium becomes thickened or blocked as a result of plaque aggregation in the epithelium [97]. The autonomic nervous system (ANS) regulates a range of functions, including cardiovascular regulation through the heart and carotid artery baroreceptors (blood pressure valves) [98,99]. When baroreceptors detect a change in blood pressure, a signal is transmitted to specific brain locations, then the ANS transmits a signal to the heart, which regulates the heart rate and cardiac output [100,101]. Signals are also carried by the arteries, causing them to spasm and regulate blood pressure [102]. CVD and PD are influenced by advanced age, diabetes, and gender. Dysregulation of the ANS can be caused by glucose metabolism, inflammation, cellular stress, or lipid metabolism [103,104]. The most common medical problem among older patients is stroke [105]. However, studies investigating the relationship between PD and stroke have shown conflicting results [106,107]. Strokes, such as cerebral ischemia, usually coincide with PD pathology according to postmortem investigations, and those clinically diagnosed with PD commonly have inflammation and fibrosis [21].

Patients with PD who are exposed to, (i) a cold environment, (ii) muscular activity in the early morning, (iii) standing positions, and (iv) over age, all have much higher sympathetic neuronal discharges, which leads to an increased myocardial oxygen requirement [108,109]. In the absence of a distinct clinical state, autonomic alterations cause homeostasis [110,111]. Thus, coronary artery stenosis demands more oxygen consumption and more coronary blood flow, but due to its shortage of blood flow and blood restriction, it results in ischemia and arrhythmias [112,113].

**Figure 6:**
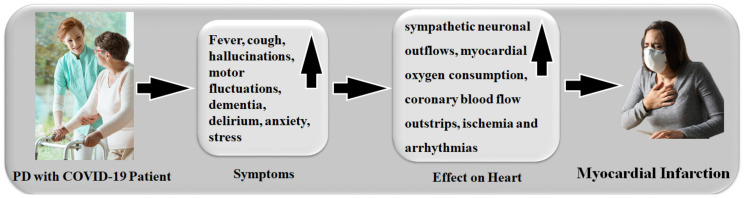
A risk factor in PD with COVID-19 patients responsible for myocardial infarction (Courtesy of AtheroPoint, Roseville, CA, USA).

People with PD and a COVID-19 infection have a risk factor that causes myocardial infarction, as shown in Figure 6. The metabolic syndrome is always linked to a group of cardiovascular risk factors, such as abdominal obesity, high blood pressure (EBP), dyslipidemia, and low blood sugar. All of these factors are linked to CVD and a higher risk of death from CVD and other causes [114]. Table 1 covers several characteristics related to the relationship between PD and CVD without the COVID-19 effect.

### 4.2. The Relationship between Parkinson’s Disease and Stroke without COVID-19

Stroke is one of the major causes of mortality in patients with PD [115,116], and this is often hemorrhagic stroke [20,117]. It is also the most common reason for long-term disability in PD patients [93,104]. Due to disturbance in the cerebral blood flow, there is an initiation of neuroinflammatory cascades that can impair the brain metabolism, which in turn leads to neuronal death [118,119,120]. Additionally, motor difficulties in PD patients lead to patient falls, resulting in traumatic brain injury. These are both major factors related to stroke risk [121].

Levodopa (also known as L-dopa) is the best treatment for PD [122]. Homocysteine levels have been reported to rise with the usage of L-dopa [78]. The pathogenic process of O-methylation of L-dopa to 3-O-methyldopa is linked to S-adenosyl methionine conversion to S-adenosyl-L-homocysteine and then homocysteine [123,124]. Patients with PD who take L-dopa and homocysteine have an increased risk of cardiovascular issues [125]. The most hazardous side effect of L-dopa is ventricular arrhythmia, although uncommon in a healthy heart, myocardial irritability and ischemia pose a serious threat to people who have them [126]. Patients with a history of cardiac abnormal activity should be approached with caution and monitored electrocardiographically [127]. Table 2 shows the link between stroke and PD in the absence of COVID-19. Stroke, traumatic brain injury, and heart rate variability [128] are all linked to PD in the vast majority of research.

### 4.3. The Relationship between Parkinson’s Disease and COVID-19

**Figure 7:**
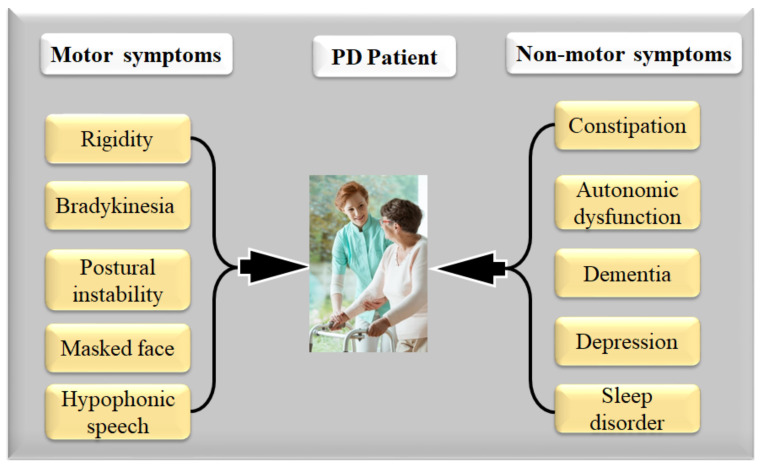
Motor and non-motor symptoms in PD patients with or without COVID-19 (Courtesy of AtheroPoint™, Roseville, CA, USA permission granted).

**Figure 8:**
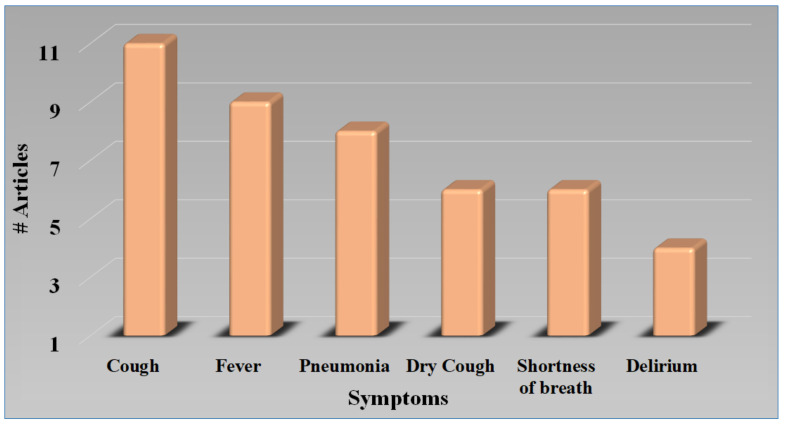
The symptoms of COVID-19 in PD patients.

The COVID-19 infection had quickly spread over the world since December 2019, resulting in a worldwide coronavirus pandemic in 2020 [7,129]. PD is a relatively common chronic illness among the elderly. Figure 7 shows the various motor and non-motor symptoms observed in PD patients with or without COVID-19. Fever, coughs, autonomic dysfunction, diarrhea, fatigue, and other symptoms have been recorded across several investigations as common COVID-19 clinical symptoms. COVID-19 also showed typical laboratory findings and irregularities on chest CT scans [130]. Figure 8 shows the symptoms of COVID-19 in PD patients. As seen in the graph, the cough is the most severe symptom, leading to upper respiratory tract infection, thus PD patients with COVID-19 have more severe lung lesions [4,131,132].

**Table 2:** Parkinson’s disease leading to stroke without COVID-19.

SN	Citations	PS	ME	Relation *	Outcome	TRE
1	Li et al. [112] (2018)	63	LBBM	Stroke and CAD in PD	When it comes to reducing the risk for heart disease, exercise may be useful in some cases. It has been discovered that having high amounts of blood cholesterol, smoking cigarettes, and having a high BMI are all connected with the development of PD.	NR
2	Studer et al. [133] (2017)	73	LBBM	Heart-rate variability and skin resonance in PD	Both SSR and HRV tests are effective in detecting ANS failure in PD patients, not only in the later stages but also in the early stages. Patients with PD may benefit from utilizing these tests to rule out autonomic dysfunction.	NR
3	Liu et al. [134] (2014)	32	Self-reporting	Stroke in PD	Since cerebrovascular and neurodegenerative diseases coexist, cerebral infarction is linked to PD. However, even though levodopa raises homocysteine levels, it is the most effective and required symptomatic treatment for many PD patients.	NR
4	Becker et al. [20] (2009)	NR	LBBM	Risk of stroke in PD	Homocysteine levels that are too high in people who have PD may make them more likely to have a stroke. There has been a link between high levels of homocysteine and a higher likelihood of stroke and heart disease. Vascular disease and dementia, as well as a rise in homocysteine levels in the blood after taking levodopa, are some of the side effects.	NR
5	Levine et al. [105] (2009)	NR	LBBM	Traumatic brain injury in PD	Patients with neurological problems can benefit from exercise training by feeling less physically and mentally worn out all the time. People with PD who engage in cardiovascular activity report less fatigue as a result of their efforts.	NR
6	Rickards [135] (2005)	NR	NR	Stroke in PD	Patients with chronic neurological illnesses are more likely than the general population to experience debilitating depressive symptoms. It is unclear what causes them, but they may be multifactorial in some cases.	NR
7	Mastaglia et al. [136] (2002)	100	Self-reporting	Prevalence of stroke in PD	Findings were not directly compared with those of prior investigations of stroke-related mortality and morbidity in the PD group following postmortem examination.	NR

* SN: serial number, PS: patient size, ME: method of evaluation, Relation: effect of PD on stroke, NR: not reported, SSR: sympathetic skin response, HRV: heart rate variability, OH: orthostatic hypotension, LB: lab-based.

Several studies have shown the effect of COVID-19 on other comorbidities such as cardiovascular stroke [36,137], brain and heart injury [54], acute respiratory syndrome [50], pulmonary embolism [138], pneumonia [132], diabetes [8,139], prediction of coronary artery disease [140], thyroid cancer detection [44,141], and liver [33], prostate [142,143], and ovarian cancers [42,144] results in worsening the symptoms of the diseases and more complications in patients, resulting in a high mortality rate.

**Figure 9:**
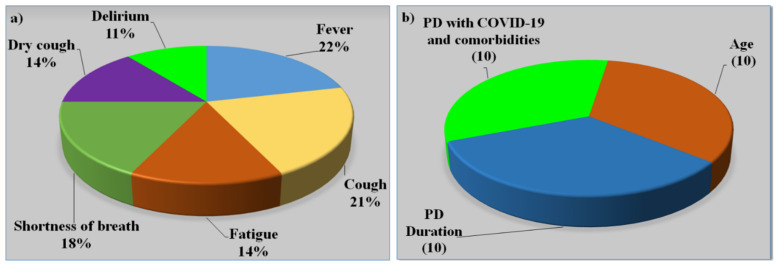
(**a**) The symptoms in patients with joint PD and COVID-19. (**b**) Risk Factors of PD and COVID-19 with comorbidities.

COVID-19 significantly exacerbated both motor and non-motor symptoms in PD, according to the current study, however, cognitive functioning was only minimally influenced [7]. Figure 9a represents various symptoms in PD patients. In terms of vulnerability, PD might be regarded as a high risk for infection, indicating the involvement of the respiratory system, which is frequently in the area of bradykinesia [130]. COVID-19-positive PD patients are more likely to be overweight, possess severe COPD, and not take vitamin D supplements than COVID-19-negative PD patients [145]. The negative correlation between COVID-19 and vitamin D supports the hypothesis that hypovitaminosis D may be a contributing factor to COVID-19 susceptibility [146]. In other groups, obesity and respiratory disease are well-documented risk factors for heart disease and stroke [147], and the negative relation with vitamin D continues to support the suspicion that iodine deficiency may contribute to COVID-19 susceptibility. Vitamin D insufficiency is frequently found in people with PD [148], and some researchers have suggested that vitamin D treatment might protect people against both COVID-19 and PD [149]. The association between PD and COVID-19 is depicted in Table 3. The majority of research makes observations about the size of the PD/non-PD dataset, its demographics, and aligned comorbidities. PD is always associated with comorbidities along with high age [130,150]. Figure 9b shows the risk factors of PD and COVID-19 with comorbidities [6,129].

### 4.4. Effect of Comorbidities on Parkinson’s Disease

This section explains the role of various comorbidities that trigger the motor and non-motor symptoms of the PD patient, and whether the patient falls under the high-risk category. Dementia develops when neurons die, causing chemical changes in the brain [11,12]. In the literature, PD is always associated with comorbidities along with high age. The long duration of PD falls under the high-risk categories [19,24,130,134]. In terms of vulnerability, PD might be regarded as a high-risk condition for infection, indicating the involvement of the respiratory system, which is frequently in the area of bradykinesia [130].

Depression is a mental illness that can decrease a human’s capacity to carry out everyday tasks, and anxiety affects around half of the people with PD [151]. This is regarded to be separate from being depressed as a result of their illness. Depression, like uncontrollable shaking, is thought to be a sign of PD. Both are brought on by changes in brain chemistry [9,10].

Persons with PD have a higher chance of having TMD than people without PD [152]. In most cases, the disc inside the jaw area moves out of position. The muscles in the joint are essentially ‘pinched’ by the slipped disc, causing them to transmit odd signals to the brain, resulting in tics or shaking [13,153]. As the dentures wear out or are removed, the jaw bone collapses into the jaw joint, which is common in older adults having PD [154,155].

People with PD have trouble passing urine since their bladders will not contract as they should. Furthermore, their sphincter muscles don’t allow urine to pass out [156]. This is due to low levels of dopamine affecting the bladder’s movement effectiveness, resulting in a residual quantity of urine remaining in the bladder [157]. This lowers the bladder’s total capacity and makes it feel as if it has to be emptied often. Unfortunately, if the bladder is not emptied, there is an increased chance of CKD [156,158]. The main cause of that disease is neurodegeneration, which damages the brain cells. The symptoms of PD and Alzheimer’s disease are dementia, anxiety, fear, and sleeping problems [151]. Neurological diseases can cause hallucinations and delusions, which are psychiatric symptoms [159]. COVID-19 considerably increases motor and non-motor symptoms in PD.

**Figure 10:**
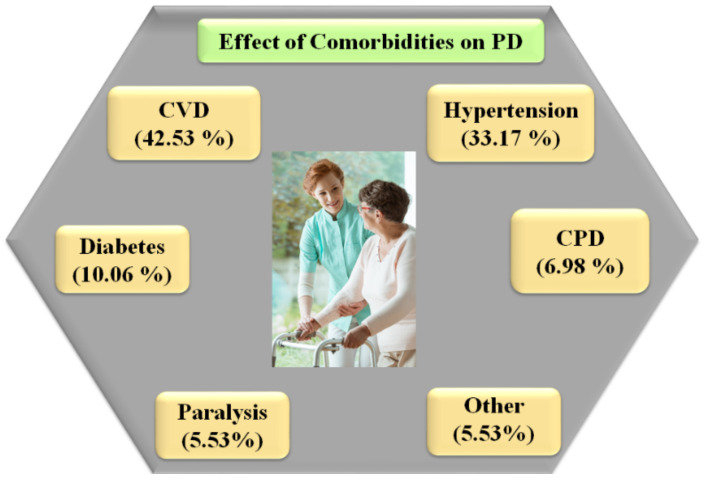
Effect of comorbidities on PD with or without COVID-19 [162].

Osteoporosis and osteopenia are common among PD patients, with women experiencing the condition at a higher rate than men [160]. Decreasing movement in PD appears to be the primary cause of decreased bone density, a process similar to that seen in other neurological illnesses [161]. Figure 10 shows hypertension (33.17%), CPD (6.98%), paralysis (5.53%), cerebrovascular disease (42.53%), and diabetes (10.60%) were the most common comorbidities among PD patients [162].

**Table 3:** Studies showing the effect of COVID-19 on PD.

SN	Author	Year	Demographics	Age	Sex	Type	Data Size	Non-PD	PD	PD w/s COVID	PD Years	Gold Standard
1	Antonini et al. [56] (2020)	2020	European	68	MF	PD with COVID	10	0	10	10	20	PD + COVID-19 + Respiratory dysfunctions
2	Baschi et al. [7] (2020)	2020	European	60	MF	PD with COVID	34	0	34	34	6	PD + COVID-19 + Pneumonia
3	Brown et al. [163] (2020)	2020	European	70	MF	PD with COVID	102	40	62	51	4	PD + COVID-19 + Respiratory dysfunctions
4	Cella et al. [2] (2020)	2020	European	65	MF	PD with COVID	141	0	12	12	4	PD + COVID-19 + Respiratory dysfunctions
5	Starmbi et al. [129] (2021)	2021	European	65	MF	PD with COVID	105	0	32	32	4	PD + COVID-19 + Pneumonia
6	Helmich et al. [6] (2020)	2020	European	NR	NR	PD with Coved	NR	NR	NR	NR	NR	PD + COVID-19 + Respiratory dysfunctions
7	Khoshnood et al. [5] (2021)	2021	European	NR	NR	PD with COVID	NR	NR	NR	NR	NR	PD + COVID-19 + Pneumonia
8	Lau et al. [16] (2021)	2021	European	NR	NR	PD with COVID	NR	NR	NR	NR	12	PD + COVID-19 + Respiratory dysfunctions
9	Sulzer et al. [4] (2021)	2021	NR	NR	NR	PD with COVID	NR	NR	NR	NR	NR	PD + COVID-19 + Respiratory dysfunctions
10	Tsivgoulis et al. [131] (2021)	2021	NR	NR	NR	PD with COVID	NR	NR	NR	NR	6	PD + COVID-19 + Pneumonia
11	Sorbera et al. [130] (2021)	2021	European	65	MF	PD with COVID	18	5	13	9	3	PD + COVID-19 + Pneumonia

### 4.5. The Relationship between Combined Parkinson’s Disease and COVID-19 on CVD/Stroke

PD patients with underlying diseases like CVD, diabetes mellitus, and hypertension are more vulnerable due to COVID-19 as it increases cardiac events [164]. Meanwhile, in PD the automatic control of the cardiovascular system is disrupted for two primary reasons. First and foremost, Lewy bodies are usually seen in the brain regions that govern the system, and these regions have also suffered from neurodegeneration [133]. Furthermore, inclusions resembling Lewy bodies and neurodegenerative diseases have a direct impact on the ANS [165]. Therefore in some cases, an attempt to raise blood pressure by the carotid artery and the heart’s baroreceptors is unsuccessful because the signals are not received [15]. As the ANS malfunctions excessively, this results in neurogenic orthostatic hypotension (nOH) or a fall in blood pressure when the PD patient walks [14,15].

Many reports regarding hospitalized patients have indicated that 12% to 26% of them had suffered heart damage. The cytokines generated during the COVID-19 infection may affect the patients’ intracellular coronary arteries. In individuals with COVID-19-affected lungs, cardiovascular illnesses have a significant impact on the ARDS [166]. These processes that lead to SARS-CoV-2 might cause a susceptible plaque to become complex and burst [55].

ACE2 receptors are highly expressed in dopaminergic neurons and are lowered in PD due to the degenerative changes [167]. Central nervous system penetration caused by the acute respiratory syndrome, SARS-CoV-2, may cause considerable harm, worsen illnesses, and increase the need for dopamine hormone treatment [168]. In many infected patients, the COVID-19 virus’s capacity to enter the brain through the nasal cavity causes anosmia/hyposmia and ageusia, addressing the variations which closely mirror one of the most notable premotor symptoms of PD [2], neurodegeneration as SARS-CoV-2 promotes the accumulation of alpha-syncline (aSyn), the major protein component of Lewy bodies in the brain [163,169,170]. Pathways impacted by a viral infection, like proteostasis, are important in maintaining dynamic equilibrium and activating stress response mechanisms, which appear to be targeted in neurodegenerative processes [163].

**Figure 11:**
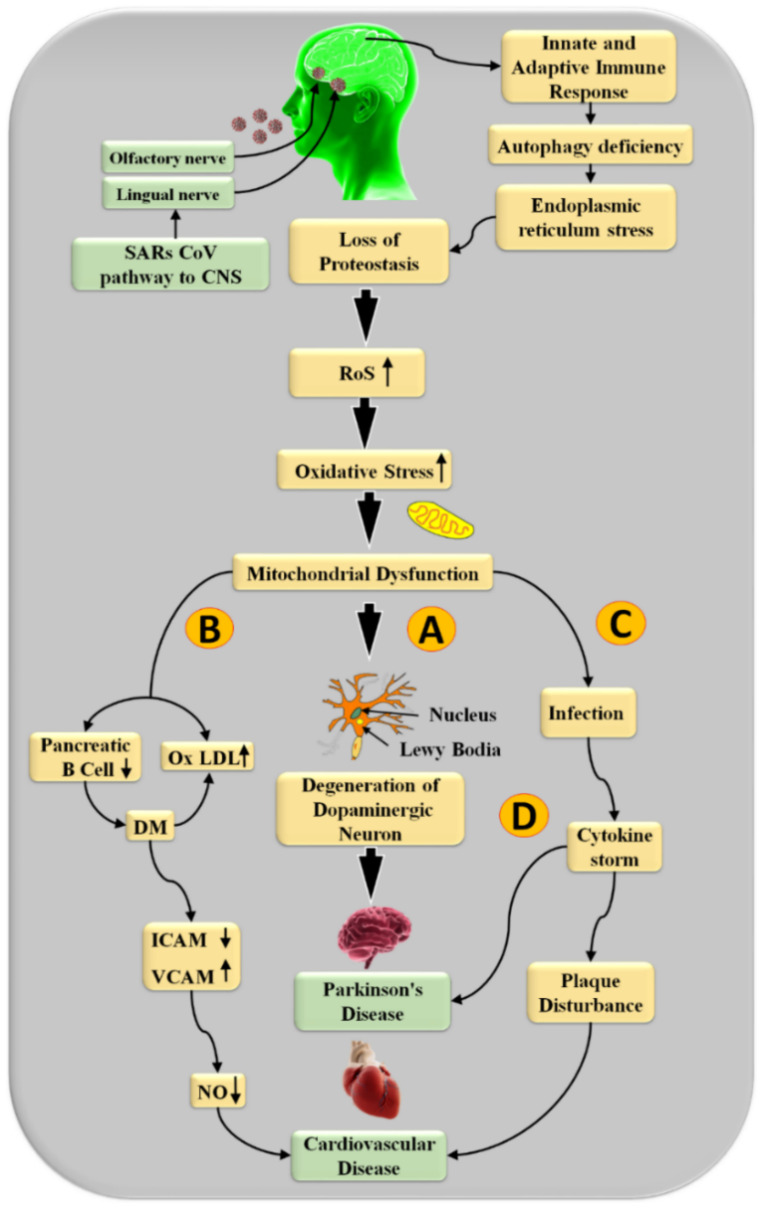
COVID-19 virus pathways leading to stroke and CVD in PD patients (Courtesy of AtheroPoint, Roseville, CA, USA).

The biochemical link between PD with COVID-19 and CVD is seen in Figure 11. It has been observed that as the infection of SARS-CoV-2 takes place through the central nervous system (CNS) and in the basal ganglia region of the brain, it triggers ACE-2 enzymes, which results in the adaptive immune response that leads to autophagy deficiency, endoplasmic reticulum stress, and loss of proteostasis [56,130].

Oxidative stress has been proven in several studies to be the most essential contributor to the development of PD that leads to CVD [79,171]. Mitochondrial dysfunction is promoted by excessive generation of reactive oxygen species (ROS) [172]. Nevertheless, as Yu et al. [173] and Bennett et al. [174] showed, it also stimulates the progression of atherosclerosis through multiple methods. Furthermore, as seen in Figure 9, mitochondrial dysfunction causes PD and cardiac injury via four separate pathways.

The substantial nigra’s selective loss of dopaminergic neurons involves oxidative stress as a critical stage [175] and is explained by path (A) of Figure 11. Resting tremors, stiffness, and balance problems are the three main signs of PD [176]. Oxidative stress damages beta cells in the pancreas and promotes the growth of oxidative lipoprotein oxidation (OxLDL) in route (B). This causes endothelial dysfunction in arteries [177]. Inhibited endothelial cell intercellular adhesion molecule (ICAM) and vascular cell adhesion molecule (VCAM) levels increase the stickiness [178]. These reduce nitric oxide (NO) levels, which support the development of the atherosclerotic plaque [179]. Furthermore, route (C) and (D) depict the link between mitochondrial dysfunction leading to infections and generating the cytokine storm that leads to plaque disturbance, which is a substantial joint risk factor for PD and cardiovascular disease [180,181]. Excess ROS and mitochondrial impairment are both involved in the pathophysiology of PD and CVD [182]. The US is the most widespread technique used, it is simple to use, has a high resolution, is cost-effective, and is a user-friendly image collection modality for plaque detection [183]. As a result, it has a broad use for regular atherosclerotic plaque monitoring and CVD risk analysis [184,185].

## 5. Deep Learning for CVD/Stroke Risk Assessment in PD Patients with COVID-19

To stratify an early CVD/stroke risk in PD patients embraced by the COVID-19 framework, AI is the most promising and optimal solution due to its ability to handle non-linearity during the training process [186]. The class of AI was first dominated by the ML systems consisting of a variety of applications, including diabetes [139,187,188], neonatology [189], genetics [190,191], coronary artery disease risk stratification [140,192], classification of carotid plaques [193], and cancer risk stratification in organs such as the thyroid [39,194,195], breast [196], ovary [142,197], and prostate [144,198], to name a few. These methods have generic drawbacks such as ad hoc feature extraction during the training/prediction design.

DL has been shown to have penetrated all walks of life and more recently into healthcare imaging [199,200]. A deep neural network (DNN) is a class of DL that mimics the human brain [32]. DL uses the power of convolution, max pooling, and different kinds of channels such as attention maps including spatial and temporal, to automate the feature extraction, classification, and segmentation paradigms [201,202]. Many studies have already described the use of AI in the diagnosis and prediction of PD [137,157,203,204,205] or prediction of early COVID-19 lesions [49,54,129,206]. Further, DL has also played an important role in the diagnosis of COVID-19 in the presence of comorbidities, such as diabetes [36], CVD [38,184,207], rheumatoid arthritis [208], and pneumonia [150]. When such comorbidities are present in the patients besides PD and COVID-19, it severely affects the non-linear dynamics. As a result, the role of DL becomes even more important and prominent in CVD/stroke risk stratification [209]. We, therefore, need better biomarkers that can measure COVID-19 severity. One such biomarker is the COVID-19 lesion size. Section 5.1 shows the role of DL for COVID-19 CT lung lesion segmentation and quantification, while Section 5.2 shows the role of DL for CVD/stroke risk stratification in PD patients affected by COVID-19.

### 5.1. Deep Learning for COVID-19 Lesion Segmentation and Its Quantification in CT

The power of DL for COVID-19 lesion detection has been shown in previous studies using different imaging modalities [17,210,211,212,213]. In fact, the use of DL has been investigated for lesion detection in several applications, such as in, (i) common carotid artery [208,214], (ii) coronary artery [140,213], (iii) brain tumor [212], (iv) skin cancers [211,215], and (v) CT-based pulmonary imaging [132]. The pulmonary lesions during COVID-19 are caused by the single-stranded RNA virus SARS-CoV-2, which infects the human cells induced by angiotensin-converting enzyme II (ACE2), which in turn leads to interstitial damage [216]. When it comes to the lesion, the stiffness in the lung muscle can be categorized as another lesion due to PD, known as bradykinesia [52,81], where the lung muscles become weaker.

**Figure 12:**
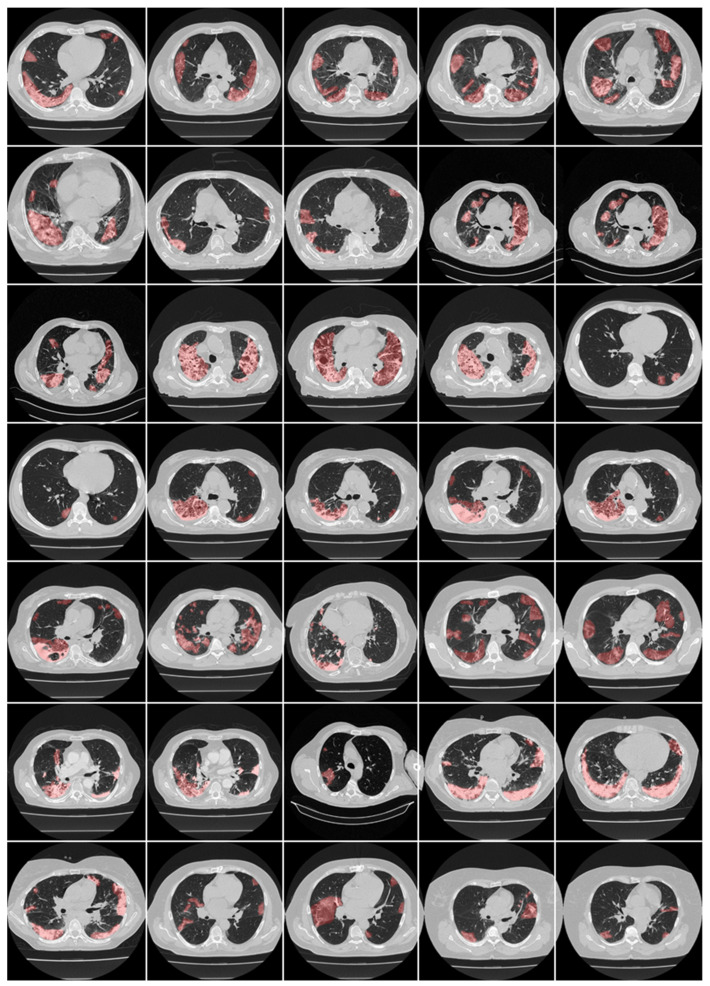
Manual lesion delineation overlays (red) from tracer 1 on raw CT lung images (Courtesy of AtheroPoint™, Roseville, CA, USA permission granted).

**Figure 13:**
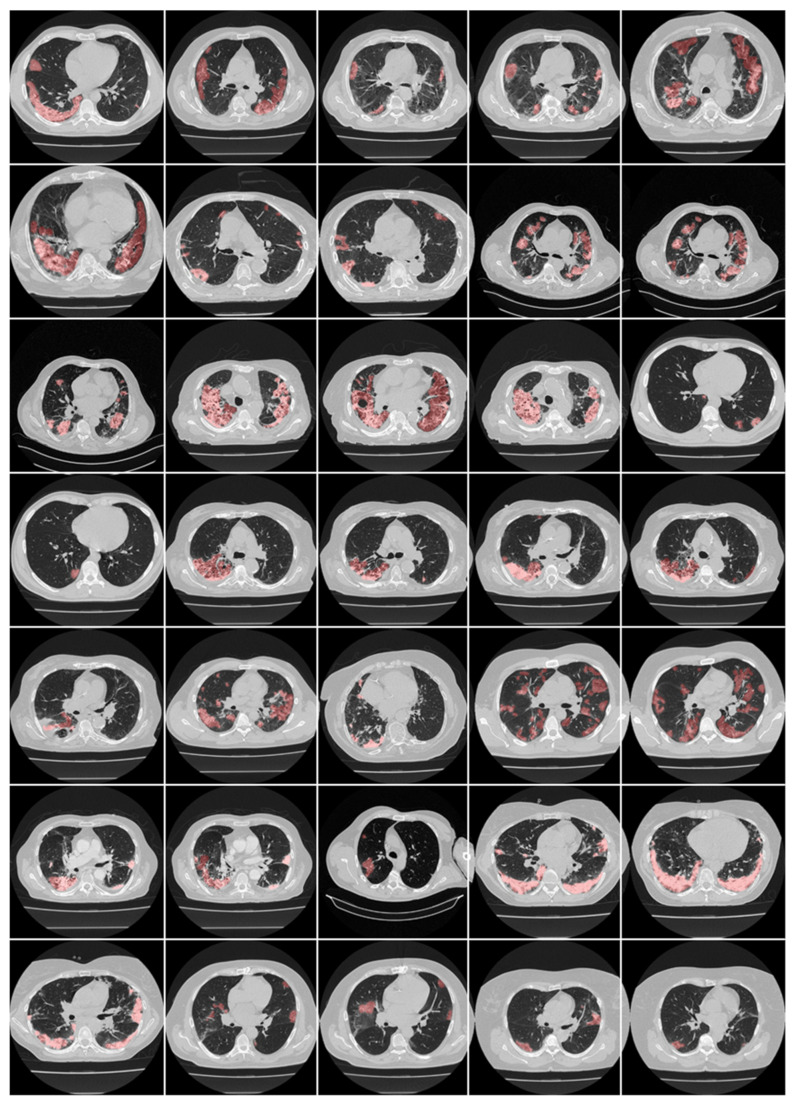
Manual lesion delineation overlays (red) from tracer 2 on raw CT lung images (Courtesy of AtheroPoint™, Roseville, CA, USA permission granted).

Here, we focus on CT-based lung lesion segmentation and its quantification, which acts as a covariate (or feature) during the DL paradigm. In DL, manual delineations of CT lung lesions are challenging and are also vital for the design of offline DL training models. Figure 12 and Figure 13 illustrate the highlighted COVID-19 lesions in CT lungs using manual delineation by experienced tracer 1 and 2, respectively. This is shown in the red color lesion as an overlay image with a grayscale CT image in the background. When it comes to COVID-19 lung lesion detection and quantification in CT, Suri et al. [217,218] have demonstrated the usage of hybrid DL (HDL) models vs. solo DL (SDL) models, exhibiting its superiority for lung lesion segmentation in CT scans. One of the most important aspects of DL is the optimization of hyperparameters during training to obtain the best performance of the DL system. It thus requires optimizing, (i) learning rate, (ii) number of epochs, (iii) batch size, and (iv) batch normalization, and (v) adding dropout layers to prevent overfitting and obtain generalization. Further, to achieve the best DL design, one must use several sources of biomarkers with a different set of data sources in a large amount in a big data framework, ensuring a multiresolution framework for faster execution time [202]. Transfer learning can also be used in CT lesion segmentation for transferring the knowledge between models, so-called pretrained models, ensuring higher speed [150,219]. Table 4 shows a variety of pretrained DL models such as DenseNet 201, ResNet50 V2, MobileNet, and VGG-16. SegNet and UNet models are stronger than the CNN models. The ResNet50 V2 model has higher accuracy compared with the 3-layer CNN and VGG-16 networks.

### 5.2. Deep Learning for CVD/Stroke Risk Assessment for Joint PD and COVID-19 Patients

**Figure 14:**
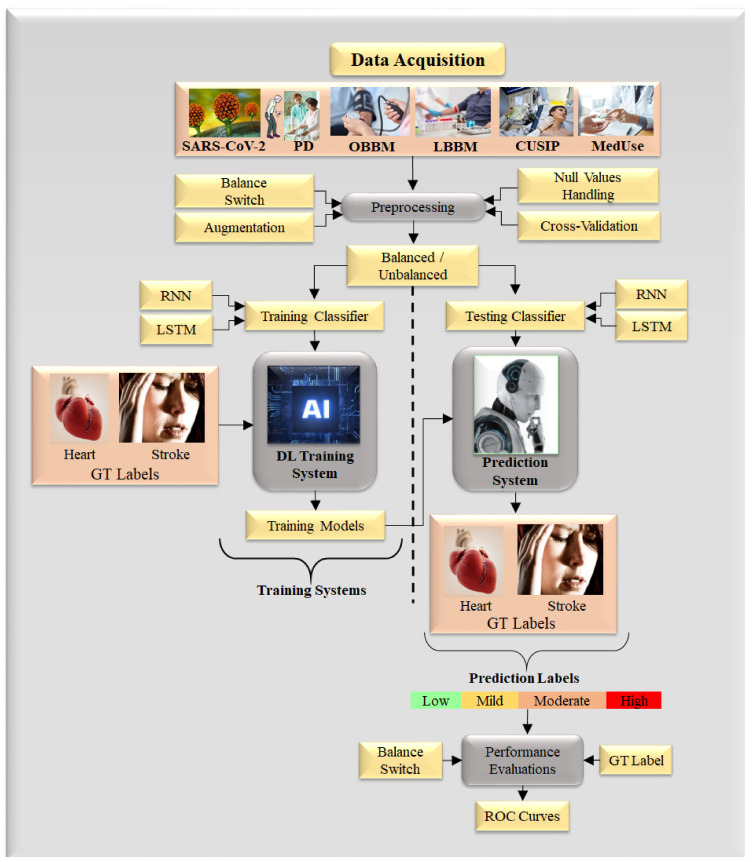
Deep learning model to predict the severity of CVD/stroke in PD with COVID-19 framework (Courtesy of AtheroPoint™, Roseville, CA, USA).

DL is a strong framework because it has the ability to derive the automated features using the inherent knowledge base and further offers a superior training paradigm where the non-linearity between covariates and the gold standard is dynamically adjusted. One such typical system for DL design is shown in Figure 14. This architecture consists of: (a) a training model design utilizing the risk variables taken from six sources such as office-based biomarkers (OBBM), laboratory-based biomarkers (LBBM), carotid image-based phenotypes (CUSIP), medication consumption (MedUSE), PD, and COVID-19, derived from the training dataset, and (b) risk prediction labels as part of the gold standard which are either heart failure (cardiovascular events) or stroke (cerebrovascular events) [220]. Such a training system can be non-linearly adjusted and has been shown recently in the context of cardiovascular risk stratification [38,185,207,221,222]. The image-based phenotypes derived from the carotid ultrasound scans are considered CUSIP [67] such as carotid intima-media thickness (cIMT, ave., max., min), intima-media thickness variability (IMTV), and total plaque area (TPA). The choice of carotid artery non-invasive imaging [48] with noise-reduction capability is preferred for economic reasons [44,223]. The carotid wall segmentation aids in the detection of plaque build-up [224,225].

The DL-based detection of PD can be conducted by using symptoms of PD as an input parameter for the algorithm. There are numerous investigations that link changes in voice [226] to a diagnosis of PD. Also, tremor [1], EEG [227], and sketch [228] biomarker data are important factors in determining if the patient has PD or not. Table 5 lists AI studies showing PD detection without COVID-19. Performance parameters of 12 studies aligned with the type of input and AI architectures. The AI-based detection of PD can be achieved by using symptoms as an input parameter for the algorithm. The majority of the studies explain voice as an input parameter for the diagnosis of PD.

**Table 5:** AI techniques and their performance for PD detection without COVID-19.

Attributes (Left to Right)	C1	C2	C3	C4	C5	C6	C7	C8	C9
Citations	IP	AI	CLS	ACC	SEN	SPEC	AUC	MCC	F1
Hoq et al. [229] (2021)	Voice	HDL	SVM	94.0	NR	NR	NR	0.71	0.91
Kamble et al. [230] (2021)	HW	ML	SVM	96.0	NR	NR	0.87	NR	0.8
Alzubaidi et al. [231] (2021)	Tremor	HDL	DT	87.9	NR	NR	NR	89.34	1.17
Khedr et al. [232] (2021)	Voice	ML	SVM	95.8	90.24	92.3	NR	92.03	96
Mei et al. [53] (2021)	Voice	ML	KNN	83.07	NR	NR	0.91	NR	NR
Singamaneni et al. [1] (2021)	Voice	ML	SVM	94.86	NR	NR	NR	NR	NR
Jayachandran et al. [233] (2020)	Voice	ML	NB	78.34	NR	NR	NR	NR	NR
Anitha et al. [234] (2020)	Voice	ML	SVM	90.21	1.8	4.39	2.49	NR	1.17
Maitín et al. [235] (2020)	EEG	ML	LR	62.99	0.9067	0.981	NR	NR	NR
Poorjam et al. [236] (2019)	Voice	HDL	SVM	96.00	NR	NR	NR	NR	NR
Aseer et al. [237] (2019)	HW	SDL	SVM	98.28	NR	NR	NR	NR	NR
Naghsh et al. [35] (2019)	EEG	SDL	DT	97.38	NR	NR	NR	NR	NR
Wang et al. [234] (2017)	BM	HDL	KNN	96.12	NR	NR	NR	NR	NR

AUC: Accuracy, SEN: Sensitivity, IP: Input parameter, AI: Artificial intelligence model, CLS: Classifier, SPEC: Specificity, MCC: Matthew’s correlation coefficient, NPV: Net present value, F1: Dice similarity coefficient; HW: Handwriting; BM: Biomarker, NR: Not reported, HW: Handwriting, SDL: Solo deep learning, HDL: Hybrid deep learning, DL: Deep learning, EEG: Electroencephalogram.

### 5.3. Deep Learning LSTM Architecture

**Figure 15:**
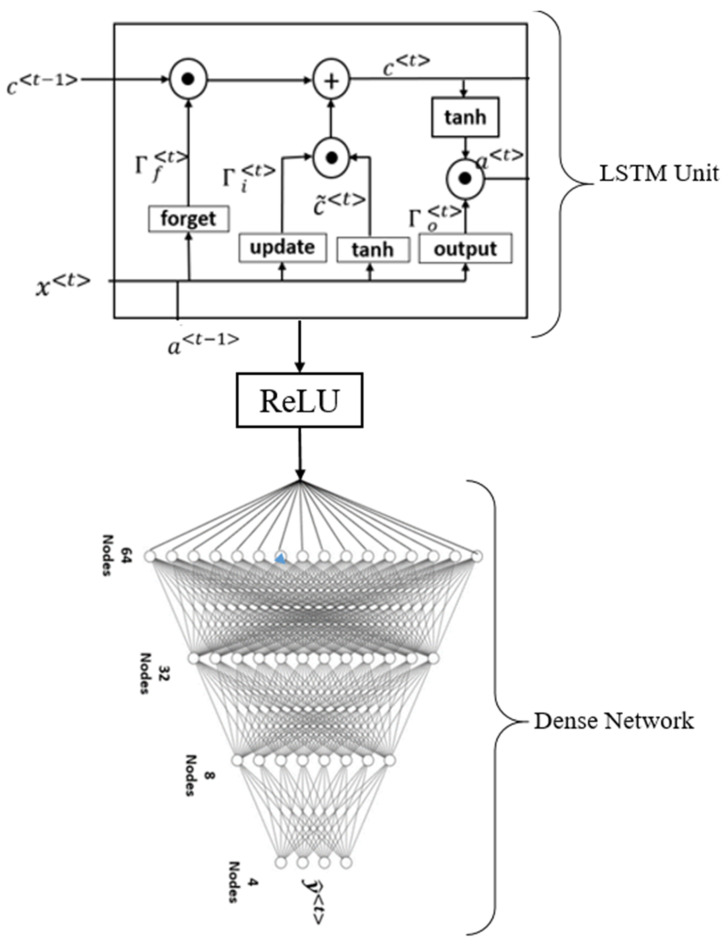
The general structure of LSTM architecture [242].

Long short-term memory (LSTM) is one of the DL algorithms that can be used to assess the risk of CVD/stroke (Figure 15). The ability to analyze several types of datapoints, such as a single observation, is the fundamental feature of LSTM. This architecture consists of four main components: cells, update gates, output gates, and forget gates (Figure 15). A cell is the central component of the design. The values are stored in the cell during random intervals, and the three gates control the flow of information or features into and out of the cell [238]. LSTM is composed of four dense layers that are fully coupled and stacked on top of one another [239,240]. LSTM performs better when it comes to formulating long-term interconnections in data [241].

### 5.4. The Comparative Analysis of AI Systems with a Different Set of Input Covariates

In previous sections, we demonstrated how patients having PD with COVID-19 increase CVD/stroke-related complications. We propose in Table 6 several AI-based studies for the CVD/stroke risk stratification of PD patients in the COVID-19 framework. The main ingredient of this table is the use of input covariates for the AI design for CVD/stroke risk stratification. Due to the addition of a large number of covariates, the non-linear dynamics increases and therefore affects the AI models during training and prediction, affecting the accuracy. We thus need robust DL-based systems for CVD/stroke risk prediction and stratification. There are six types of covariates that are used for the design of the AI models, and we call them six types of AI clusters labeled as: (i) OBBM, (ii) LBBM, (iii) CUSIP, (iv) MedUSE, (v) PD, and (vi) COVID-19. Note that the gold standard in all AI design solutions while considering these covariates are patients who had a myocardial infarction, coronary artery syndrome, coronary artery stenosis, or stroke. These clusters are discussed below:

(i) AI systems that use office-based biomarkers as input covariate

All the studies in Table 6 use OBBM as an input covariate, and this consists of attributes such as height, weight, BMI, gender, ethnicity, smoking status, hypertension, and cholesterol levels. An example of OBBM use is Kakadiaris et al. [243], where the authors proposed an ML-based risk calculator for a multiethnic, community-based population of men and women examined for incidental atherosclerotic CVD. The authors employed ACC/AHA risk assessment variables and had an ML accuracy of 86%.

Reva et al. [244] described the first AI-based algorithm for CVD risk assessment capable of reliably monitoring the collateral flow in androgen insensitivity syndrome (AIS) patients. This is an automated technique that reduces bias and streamlines the clinical process, which helps in determining reperfusion-eligible patients. Collateral circulation is connected to a better functional outcome in acute ischemic stroke patients with major arterial occlusion. Due to complex neuro-vasculature, evaluating collateral flow can be difficult and time-consuming. The authors adopted SVM and RF-based ML algorithms for the classification of AIS patients vs. controls. The model used 300 patient data and reported an accuracy of 87%.

(ii) AI systems that use laboratory-based biomarkers as input covariate

Biomarkers are chemicals released into the blood by a damaged or stressed heart. These indicators are used to diagnose acute coronary syndrome and myocardial ischemia. Cardiovascular biomarker tests can also be used to assess a patient’s risk of developing CVD or heart ischemia. There are various LBBM related to the heart such as low-density lipoprotein, high-density lipoprotein, myoglobin, creatine kinase, troponin, atrial natriuretic peptide, etc. For example, the study by the Unnikrishnan et al. [245] described a method for assessing CVD risk associated with health indicators, many of which are derived from the Framingham risk score. These approaches, however, have major limitations as a result of their low sensitivity and specificity. The study reported a cohort size of 3665 patients for studying the effect of model training on the local database, computed the Framingham score, and established the linear regression analysis. The study presented an AI model and reported an accuracy of 83%.

(iii) AI systems that use carotid ultrasound image phenotype as a covariate

**Figure 16:**
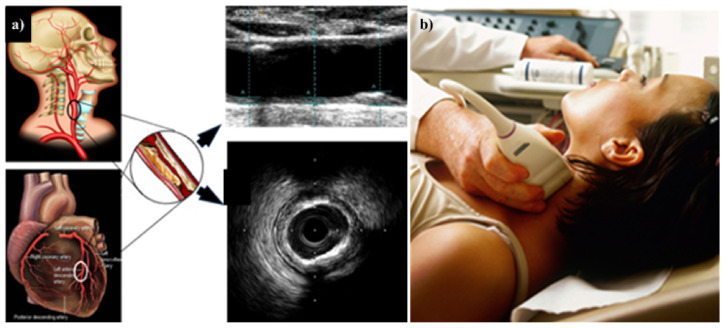
(**a**) Carotid artery disease is being investigated as a potential surrogate marker for coronary artery disease. (**b**) Imaging device where the carotid artery is being scanned with the linear ultrasound probe. The middle panel shows the B-mode carotid longitudinal US scan and IVUS-based artery cross-sectional scan [250].

In order to perform a comprehensive risk assessment, we must be able to automatically and precisely quantify CUSIP [246], which consists of carotid intima-media thickness, average, maximum, and minimum (cIMTave, cIMTmax, and cIMTmin), carotid intima-media thickness variability (cIMTV), morphological total plaque area (mTPA), geometric total plaque area (gTPA), lumen diameter (LD), and inter-adventitia [247]. We need a risk assessment system that can determine the severity of coronary artery disease in patients who present to the emergency department. All emergency department examinations discovered an increase in cardiovascular disease, which was found to be associated with an increase in phenotypes such as cIMT, gTPA, mTPA, and CRS. This CUSIP is then used as a covariate in the ML algorithm to further improve the results (Figure 16) [246]. Suri et al. [248] explained the risk of CVD/stroke in PD patients by using carotid artery imaging, since it was low-cost, non-invasive imaging for the screening. PD patients will benefit from the adoption of this low-cost B-mode ultrasonography since it will allow for the characterization of plaque tissue. This will provide a critical additional understanding of CVD/stroke risk stratification in PD patients.

(iv) AI systems that use Parkinson’s disease symptoms as input covariate

The PD input covariates are voice, gait, sketch pattern, and abnormalities in EEG. The motor and non-motor symptoms result in a better understanding of whether patients have PD or not. Another study by Park et al. [249] used EEG as an input to predict the stroke severity in the PD patients. It implemented the SVM algorithm for the classification. The cohort size consisted of only 16 patients, which was relatively small. The study reported an accuracy of 88%.

(v) AI systems that use COVID-19 as input covariate

The DL algorithm is used by the vast majority of AI-based systems for COVID-19 identification and categorization. To improve the amount of COVID-19 data available for training, some researchers have adopted data augmentation techniques. Scientists must modify the number of convolution layers in accordance with their intuition when working with this hyperparameter. However, for COVID-19, DL-focused AI systems must be adjusted based on the link between enhancement, the number of convolution layers, and classification accuracy. The study by Suri et al. [54] presented an ML model that can be used to predict the severity of CVD/stroke in COVID-19 patients. In these review studies, the authors have validated their findings that COVID-19 causes damage to the brain and heart through four distinct pathways (i.e., neuronal, hypoxia, RAAS, and immunological). The degree of risk linked with a patient’s symptoms, and invasive imaging techniques, whether portable or non-portable, must be performed with the utmost care. Even though medical imaging [251] can considerably improve a patient’s odds of survival, the shortage of qualified radiologists prevents it from being widely utilized. Furthermore, a study by Zimmerman et al. [252] explained the significance of comorbidity appears to be associated with adverse outcomes in COVID-19 patients. The uses of AI, particularly ML, have the potential to utilize data-rich platforms and alter methodologies in the diagnosis, risk stratification, prevention, and treatment of CVD. The patient size used was 32. The LDA method was used for extracting features. The CNN algorithm was used for classification purposes and the study reported an accuracy of 87%.

Another study by Handy et al. [253] explained the mortality rate prediction of CVD/stroke in COVID-19 patients. The study explained the atrial fibrillation parameter for the benchmarking of the stroke risk prediction method. The DL-based LSTM algorithm was used for the analysis purpose. The study reported an accuracy of 84%. Another study by Bergamaschi et al. [254] determined the significance of serial ECG abnormalities in hospitalized individuals with COVID-19. These findings showed the role of ECG abnormalities were detected at admission and even more, were observed at the 7-day ECG, which could assist doctors in stratifying the risk of significant adverse events in COVID-19. The severity of the SARS-coronavirus-2 infection was found to be linked to changes in the ECG. To our knowledge, no AI study has been able to give clear and useful information related to the CVD/stroke risk stratification of PD patients in the COVID-19 paradigm.

Hence, we hypothesize that DL models are capable of performing a specific task, such as automated disease diagnosis, with greater precision and efficiency than ML models and act as a second level of confirmation for the diagnosis [255]. Models trained with deep learning can be applied to a wide variety of problems, including image-based quantification, improvements to image acquisition, and differential diagnosis.

Table 6 shows the comparison between the proposed ML/AI algorithms and similar techniques for CVD/stroke risk prediction. There were published techniques available in the literature. There are other AI-based CVD/stroke and non-AI-based CVD/stroke risk stratification methods that use carotid-based biomarkers with conventional risk factors. AI-based methods have used ML techniques with conventional biomarkers or a combination of conventional biomarkers with carotid-based image phenotypes [38,184,185,207,209,221,256,257]. The main concept behind these studies was to add the covariates such as cIMT (average, max., min), IMTV, and TPA along with conventional biomarkers such as A1c, LDL, HDL, triglycerides, SBP, DBP, BMI, and age. The system designed used standardized classifiers for training when using the cross-validation approaches. For prediction system design, the test data was adapted where the training model transformed the test features. These methods are mainly called the class of AtheroEdge™ 3.0 system designs (AtheroPoint, Roseville, CA, USA). In the non-AI-based methods for CVD/stroke the risk was determined by computing the digital total of all the normalized risk values for the image-based biomarkers and then compartmentalized into different risk classes such as no-risk, low-risk, low-moderate risk, high-moderate risk, low-of-high risk, and high-of-high risk. This was computed using the AtheroEdge™ 2.0 system (AtheroPoint, Roseville, CA, USA) [36,220,221,258,259,260,261,262,263,264,265]. Image-based biomarkers, such as TPA, have shown to have a strong link with eGFR [266], and thus AI-based solution have adapted the usage of TPA in the modeling process. AtheroEdge systems were designed to keep both AI-based and non-AI-based methods at a low cost [267]. Note that the importance of the automated biomarker guidelines were recently revisited for CVD/stroke risk stratification [265], thus the above AI-based and non-AI-based methods are powerful solutions for CVD/stroke risk assessment.

**Table 6:** Comparative analysis of AI-based studies with CVD/stroke risk stratification of PD patients in the COVID-19 framework.

SN	Citations	Year	Input Covariates						GT	PS	AI	FE	CLS	ACC %	AUC
			OBBM	LBBM	CUSIP	MedUSE	PD	COV							
1	Yan et. al. [268]	2019	✓	✓	✕	✓	✕	✕	CVD	NA	NA	NA	NA	NA	NA
2	Park et al. [249]	2017	✓	✓	✕	✕	✓	✕	Stroke	18	ML	RF	SVM	88.00	NR
3	Suri et al. [248]	2022	✓	✓	✓	✕	✓	✕	CVD/stroke	NR	ML	NR	NR	NR	NR
4	Zimmerman et al. [252]	2020	✓	✓	✕	✕	✕	✓	CVD	32	DL	LDA	CNN	87.23	NR
5	Aljameel et al. [269]	2021	✓	✓	✕	✕	✕	✓	CVD/stroke	287	ML	KNN	SVM	95.00	0/99
6	Suri et al. [54]	2020	✓	✓	✓	✕	✕	✓	CVD/stroke	NR	ML/DL	NR	NR	NR	NR
7	Handy et al. [253]	2021	✓	✓	✓	✕	✕	✓	CVD/stroke	NR	ML/DL	LSTM	SVM	84.00	NR
8	Unnikrishnan et al. [245]	2016	✓	✓	✕	✕	✕	✕	CVD	3654	ML	LR	SVM	83.00	NR
9	Mouridsen et al. [270]	2020	✓	✓	✕	✕	✕	✕	Stroke, MRI	16	DL	NR	KNN	74.00	0.74
10	Bergamaschi et al. [254]	2021	✓	✓	✕	✕	✕	✕	CVD	237	NA	NA	NA	NA	NA
11	Reva et al. [244]	2021	✓	✓	✕	✕	✕	✕	Stroke, CT	200	ML	NB	DT, RF, SVM	85.32	NR
12	Kakadiaris et al. [243]	2022	✓	✓	✕	✕	✕	✕	CVD	6459	ML	DT, RF	SVM	86.00	0.92
13	Proposed study	2022	✓	✓	✓	✕	✓	✓	CVD/stroke	NA	NA	NA	NA	NA	NA

IC: Input covariate, COV: COVID-19, PD: Parkinson’s disease, CVD: Cardiovascular disease, AI: Artificial Intelligence, OBBM: Office-based, LBBM: Laboratory-based, CUSIP: Carotid ultrasound image phenotype, MedUse: Medication, GT: Ground truth, PS: Patient size, FE: Feature extraction, CLS: Type of classifier, ACC: Accuracy, AUC: Area under the curve, NA: Not applicable, NR: Not reported, ✓: Yes, ✕: No.

### 5.5. Implementation and Maintenance of AI-Based CVD Risk Stratification System

Cardiovascular disease (CVD) is the most prominent cause of global mortality and morbidity. Annually, ~17.9 million people die due to CVD, which accounts for 31% of the overall deaths worldwide. Atherosclerosis is the main cause of CVD and future cardiovascular events. Ultrasound-based carotid artery imaging is a well-established surrogate imaging for coronary heart disease and is typically adopted in office-based settings. Studies have recently shown that image-based biomarkers or phenotypes when combined with conventional risk factors are even more effective in CVD risk prediction. Such image data, when collected or derived from a cohort, can be even more useful in predicting CVD risk. Recently such an approach setup for CVD risk assessment using the combination of carotid ultrasound plaque imaging and conventional cardiovascular risk factors (such as patients’ demographics and laboratory-based parameters) in the ML framework was proposed. Granularity in expressing CVD risk is crucial for personalized medicine and better drug monitoring. Thus, one can improve the system by using multiclass–multilabel-based (MCML) algorithms by assessing carotid ultrasound imaging for predicting the presence of significant coronary artery disease than traditional risk scoring methods. Specifically, our aims were to: (i) design a carotid image-based MCML CVD risk assessment calculator, (ii) study the effect of clustering risk predictors on MCML performance, and (iii) benchmark the MCML-based calculator against three types of conventional CVD risk calculators (CCVRC) such as the Framingham risk score, the systematic coronary risk evaluation score, and the atherosclerotic CVD score.. The AtheroEdge 3.0 ML using camaging is reliable, accurate, and superior to traditional CVD risk scoring methods for predicting the CVD/stroke risk due to coronary artery disease.

(i) Implementation of Training System

For generating the training model, one needs to define the type of the training model based on the number of samples in the training system. This is done under the subsystem called cross-validation. Typically, the cross-validation is categorized by the symbol “K”. Examples of cross-validation systems are K2, K3, K4, K5, K10, and TT. K2 means 50% training and 50% testing data, K3 means 66% training data and 33% testing data, K4 means 75% training data and 25% testing data, K5 means 80% training data and 20% testing data, K10 means 90% training data and 10% testing data, and finally, TT means “Training equals Testing protocol”, where training data is 100% and testing data is also 100%. Typically, the TT protocol is adapted to validate the AI systems.

(ii) Implementation of Prediction System

This is the first study that combined conventional predictors with eight other clinical clusters of different features in an MCML using the coronary angiogram as the gold standard. Such a system can then leverage the cohort’s knowledge of nonlinearity between input predictors and the gold standard in an MCML framework by automatically and accurately predicting and stratifying the stroke/CVD risk into four granular classes. Thus, the AtheroEdge 3.0 MCML system uniquely overcomes such nonlinearity in a multiclass framework, especially by using the carotid plaque image phenotypes, providing a powerful paradigm similar to an office-based setup, where only the offline training coefficients are needed for such an accurate prediction. To address the challenge of class imbalance in the dataset, which is the most common problem in medical datasets, we used the commonly used SMOTE algorithm that generated independent samples to balance each of the minority risk classes.

(iii) Performance

Our study was highly novel and has demonstrated the superior performance of the MCML-based algorithm compared with previous similar studies. We compared our study results with five recent ML-based studies using the AUC as a common performance evaluation metric. Recently, Kakadiaris et al. [38] have presented a 13-year follow-up study with 6459 participants to predict the CVD risk using an ML classifier and compared its performance against the ASCVD risk calculators. The authors performed binary CVD risk stratification using an SVM-based ML classifier with nine conventional CVD risk predictors and follow-up cardiovascular events as the gold standard.

(iv) Maintenance

Our system works using Python, and Java in a windows 11.0 platform and for maintenance of the system, the following items are kept in mind namely: upgrade of windows platform, upgrade of open-source python system, upgrade of Java system, upgrade of the database system from Oracle, update version of the PDF distiller as needed for report generation. Furthermore, there are bug trackers as usual, which are proprietary and cannot be shared or released.

### 5.6. Distribution Strategies of the Potential Benefits of the ML/AI Model

There are several distribution strategies for the potential benefits of commercial ML/AI models. Since AI models are online systems that need “test data sets” for execution, such models can be integrated into, (i) cloud-based settings, or (ii) embedded in the scanning devices, something like “Intel processor chip in Dell computer”.

The cloud-based strategies are most effective because the system can be executed from “any computer” and “any time”. The cloud-based system can be launched in offline settings and the ultrasonic data can be physically moved from the ultrasound machines to the “local computer”. This is one way the data can be distributed to the local computer and the benefit of ML/AI can be realized.

The second distribution strategy is when the offline training models can be embedded into the scanning devices that hold the data. This kind of distribution strategy raises the price of the system, and the cost of maintenance is high due to the evolution of the ML/DL model generations. The profits are high since AI models are embedded in the system itself. If the interface of the models is smooth and the graphical user interfaces are ergonomic or user-friendly, the overall systems can be very powerful. Such distribution strategies are compact but surely encounter a higher price tag for customers. The shipping of such scanners has higher maintenance costs and is typically passed to the customers. Upgrades are more frequent due to scientific evolutions, which are again passed to the customers.

No matter which distribution strategy one chooses for benefitting the AI models, “cloud vs. embedded-based solution”, and the interface design must incorporate multi-threaded and multi-tasking architectures enveloping higher CPU clock speed, preferably GPU-based settings or even pruned models having the explainable AI-based paradigms.

## 6. Critical Discussion

In this review, we focused on the CVD/Stoke risk stratification of PD patients in the COVID-19 framework. Furthermore, it is clear from a detailed evaluation of several investigations that PD patients with COVID-19 are at an elevated risk of CVD/stroke. As a result, in addition to COVID-19 on PD patients and its monitoring, a low-cost approach should be used to prevent a patient’s CVD/stroke symptoms from worsening. With the aid of a DL-based AI model, these patients may be efficiently monitored, and long-term effects can be averted. In the presence of the COVID-19 framework, DL can assist in CVD/stroke risk stratification in PD patients, with improved sensitivity and specificity. Clinicians can use this model to counsel COVID-19-positive patients with PD along with carotid arterial imaging, and provide further guidance on the CVD/stroke risk.

### 6.1. Benchmarking

PD and COVID-19 have been linked in a few studies utilizing OBBM, LBBM, CUSIP, and MedUSE, according to an overview of the data. AI’s function in the diagnosis of joint COVID-19 and PD is rarely discussed in the literature. Only a few articles in the COVID-19 framework use the AI model to describe the severity of PD. Table 7 reports the benchmarking scheme for selected studies.

The study by Antonini et al. [56] explains that the relationship between COVID-19 and PD is fascinating due to several findings. ACE2 receptors are highly expressed in dopamine neurons and are lowered in PD due to gradual decline. Central nervous system infiltration caused by the severe acute pulmonary syndrome SARS-CoV-2 may cause additional harm, worsening ailments, and increases the need for dopamine replacement therapy, as seen in PD patients.

Baschi et al. [7] mentioned that the effects of the COVID-19 lockdown on patients with prodromal stages of dementia are unclear. The study discusses motor, cognitive, and changes in behavior in patients with PD with and without mild cognitive impairment, as well as patients with mild cognitive impairment not associated with PD. The COVID-19 quarantine is linked to worsening cognitive, psychosocial, and motor symptoms in people with PD and mild cognitive impairment. Different methodologies must be implemented to limit the effects of quarantine on patients with PD and cognitive impairment. Brown et al. [163] provided comments on COVID-19 symptoms and the pandemic’s effect on persons who had or did not have COVID-19 to quickly identify areas of need and enhance care for people with PD. The COVID-19 global epidemic has been connected to a significant impact on patients with PD, with an older age group being particularly vulnerable.

Sorbera et al. [130] explained why patients with PD are more vulnerable. As per their findings, aged people with an underlying chronic condition have a higher chance of developing severe disease or perhaps dying. PD is a common age-related degenerative disease, and it is often linked to other health problems such as cardiovascular disease, which means that PD patients are almost certain to be in a high-risk group for SARS-CoV-2 infection, because they are likely to have a lot of other health problems. In addition, the apparent association between PD, age, and cardiovascular comorbidities carries an “indirect risk”.

### 6.2. Bias in Deep Learning Systems

The training model design step of the DL algorithms is highly dependent on the sample size employed. Furthermore, due to a lack of, (i) clinical testing of AI techniques, (ii) scientific validation, (iii) not satisfying the gold standard, (iv) comorbidities, (v) lack of big data configuration, and (vi) not judging the proper disease severity ratio, these all lead to bias in the AI. As a result, when COVID-19-associated PD symptoms (or risk factors) are examined as inputs into an AI model, it is critical that the AI model be stable, accurate, and has minimal AI bias [45,224,271,272,273]. It may also be noticed that the database contains geographically specific patient characteristics. As a result, the model may produce deceptive positive or negative findings for other continents, introducing bias into the model [274,275].

### 6.3. The Economic Aspect of AI-Based Diagnosis

The field of artificial intelligence (AI) has an effect on virtually every aspect of life [32], particularly the application of machine learning and deep learning in medical imaging [32,276]. The use of AI in medical diagnostics is now in the early adoption phase across several different specialties [1]. The number of AI articles has increased exponentially. Willmen et al. [277] showed a cost-effective saving when using a referral system using decision support. One reason is the optimized, accurate, and automated solution. This directly transforms into cost benefits, leading to significant influence on the discipline of economics. Several examples have shown the cost benefits of AI in different applications of medicine.

Mital et al. [278] conducted a model-based economic evaluation that used a hybrid decision tree/microsimulation model for comparing the costs of screening mammography using eight different methods. The study showed that using AI in breast screening for low-risk women is the most cost-effective solution. Areia et al. [279] conducted the Markov model for colorectal cancer screening with and without AI. The study showed prevention of 7194 colorectal cancers along with prevention of 2089 deaths, and finally a saving of 290 million USD. According to a recent study on the internet of things (IoT), machine learning is helping to speed up the growth of industrial supply chains, which saves both time and money. Morrison et al. [280] developed a cost-effectiveness analysis of an AI-based solution [280] for retinopathy of prematurity (ROP) screening. The authors adapted the decision tree’s machine learning solutions and compared them against three different kinds of strategies such as ophthalmoscopy, telemedicine, and assistive AI with telemedicine review. The authors took a cohort of infants using ROP screening in the USA. The outcome of the study demonstrated the most cost-effective solution was using an AI-based telemedicine approach. The main reason for this was the avoidance of human examiners for detecting ROP. Bao et al. [281] showed the role of AI-based assisted reading of human papillomavirus (HPV) testing and compared it against liquid-based cytology and manually drawn readings. The study included 2065 women aged 25–64 proving their hypothesis that AI-based solutions are effective both in cost and efficiency. Hoshida et al. [282] demonstrated the cost-effectiveness of four types of hepatitis B virus (HBV) serological screening methods in China. The authors designed the Markov cohort model, by taking into consideration the parameters based on previous studies databases. Lee et al. [283] proposed an AI-based solution to faulty remote water-meter-reading (RWMR) devices. The authors adapted a convolutional neural network–long short-term memory network (CNN-LSTM) by considering 2762 customers over 360 days and collecting 2,850,000 AMI datasets in semi-rural areas of South Korea. The authors demonstrated an F-measure of 0.82 and Mathew coefficient of 0.83 using CNN–LSTM as part of the cost–benefit analysis.

Cardiovascular disease (CVD) is one of the major causes of mortalities and morbidities in the world [284]. Annually, CVD causes ~17.9 million mortalities each year [284]. From our survey, we did not find any cost–benefit analysis paper related to CVD. The early diagnosis of the risk of CVD/stroke is very important for reducing healthcare expenditures and saving lives. With AI, CVD detection can be performed early, faster, and at a low cost. Since only training models are needed that can be stored in the system, the online CVD/stroke risk prediction can be quickly done in seconds if the test patient data is available. This is highly economic since the risk stratification can be done in seconds with granularity. Rural areas are seeing a decline in the number of primary care physicians employed there. This is because rural families, on average, earn less money than families living in urban areas. Because AI can provide specific test support in place of procedures, it can help address the challenges brought about by changes in both the demographics and the economy. Now with the cloud-based internet solution, the data can be simply loaded onto the web and the system by calling the offline models to predict the risk of granularity for CVD/stroke. This saves tremendous amounts of time and, thus, eventually the cost is lower [285].

For CVD/stroke risk prediction in PD patients in the COVID-19 framework, the same set of AI benefits apply here as well. The deep learning automated model can be used for COVID-19 detection and quantification in CT scans [286]. This is fully automated and a superior time-saver, which translates into cost savings. The covariate design for ML-based CVD/stroke risk prediction is a further time-saver, since it can be used for training model one-time design. These models can then be used for CVD/stroke risk prediction on online patients, further saving time and cost. The various deep convolutional neural network architectures were explained in Appendix A.

In summary, the main attributes of economic benefits for using AI-based solutions are, (i) early detection of the disease, (ii) prevention of surgery at a delayed time, (iii) use of off-line in-built training models embedded in the systems, and (iv) Usage of cloud-based AI technologies. While AI is powerful and proving to be an economic solution, there are ethical concerns [287], lack of regulation, and handing of AI bias [216,271,272,273,288]. Further, these solutions should use big data framework [274] and blockchain framework [289] if AI is taken to its full advantage.

### 6.4. Strengths, Weakness, and Extensions

The main strength of the current system is the role of DL for CVD/stroke risk assessment in PD patients in the presence of COVID-19. DL offers better training and risk prediction due to superior non-linear adjustment between the covariates and the gold standard. Further, the system offers better coverage of covariates such as OBBM, LBBM, CUSIP, MedUSE, PD covariates, and COVID-19 covariates along with lesion sizes estimated from the CT scans of the lungs. Further, the role of LSTM, a very powerful approach for the DL system design for CVD/stroke risk prediction was presented. Lastly, the DL system is generalized which can be altered by adding more covariates and comorbidities such as diabetes, rheumatoid arthritis, renal disease, coronary artery disease, etc.

The AI-based solution for CVD/stroke risk assessment of PD patients in COVID-19 framework is the first to introduce the use of a machine learning system for CVD/stroke risk assessment and is easily amendable for adjustment of more parameters. This means more covariates can be added and the machine learning system will perform better once optimized. The deep learning paradigms are powerful solutions for lesion detection.

The first time the system is developed it computes CVD/stroke risk in PD patients in the COVID-19 framework.

While DL offers strengths to the system, it also needs to be ensured that the system is optimized always. This requires several iterations of hit-and-trial attempts to achieve optimal DL solutions. Further, the DL system requires a solid gold standard for, (a) during CT lesion annotations, and (b) CVD/stroke gold standard collection in cohorts, which also requires time and cost. Lastly, as pointed out before, DL systems are susceptible to AI bias due to overperformance in terms of accuracy and lack of interpretability. In terms of extensions, superior DL systems can be designed using ensemble-based methods. Big data can be considered as an option for improving the DL system by taking more sources of data and in a larger sample size. The DL system can be improved by adding the augmentation designs should the cohort size be small. Lastly, the new wave of pruning needs to be incorporated into the DL system for smaller-size training storage models [290] and evolutionary methods [291].

## 7. Conclusions

The importance of CVD/stroke risk prediction for PD patients in the COVID-19 environment was highlighted in this comprehensive investigation. We also illustrated how PD with COVID-19 can cause vascular and cerebral strokes. This review highlighted how PD with COVID-19 may aggravate complexity in CVD/stroke. Thus, PD patients’ CVD/stroke risk categorization in the COVID-19 paradigm is critical. Carotid imaging is a non-invasive, low-cost alternative to conventional imaging for monitoring CVD/stroke in PD patients. This low-cost B-mode ultrasonography also will help characterize plaque tissue in PD patients with COVID-19, improving CVD/stroke risk assessment. CT scan images of lung lesions can help diagnose and quantify COVID-19 infection severity, which is useable as a covariate during DL design. As a result, we explained the role of an AI-based model that can be used to accurately classify PD patients into risk groups for CVD/stroke based on their COVID-19 risk profile. The COVID-19 framework was used to describe an AI-based model for predicting CVD/stroke risk in PD patients. Finally, we discussed the involvement of joint PD with COVID-19 in the CVD/stroke paradigm, as well as the role of AI in this context.

## Figures and Tables

**Table 1:** Parkinson’s disease without COVID-19 leads to CVD.

SN	Citations	PS	ME	Relation *	Outcome	Treatment
1	Huang et al. [83] (2015)	156	LBBM	Plasma cholesterol risk in PD	Total high cholesterol levels have been linked to a lower risk of developing Parkinson’s disease, but statin use has been linked to an increased risk.	Statins
2	Yan et al. [72] (2019)	68	LBBM	Carotid plaque in PD	As Parkinson’s disease advances, the thickness of carotid plaques rises.	NR
3	Potashkin et al. [83] (2020)	47	LBBM	CVD and PD	Both CV and PD share inflammation, insulin resistance, lipid metabolism, and oxidative stress. Moderate coffee consumption and physical activity reduce the risk of heart disease and PD.	NR
4	Park et al. [35] (2020)	NR	Population-based cohort study	PD with risk of CVD	CVD is linked to PD. Patients with PD should be monitored for CVD.	NR
5	Değirmenci et al. [64]	NR	LBBM	Cardiac effect in PD	Cardiac problems are prevalent among Parkinson’s disease sufferers.	Levodopa, MOBI, COMT, anticholinergic drugs, deep brain simulations
6	Scorza et al. [84] (2018)	NR	LBBM	Cardiac abnormalities in PD	Cardiomyopathy, coronary heart disease, arrhythmias, conduction anomalies, and sudden cardiac arrest are among the symptoms of PD/PS.	NR
7	Günaydın et al. [85] (2016)	65	LBBM	CVD risk in PD under levodopa treatment	PD patients with L-dopa exhibited increased aortic stiffness and impaired diastolic performance. Homocysteine levels may influence diseases.	NR
8	Fanciulli et al. [86] (2020)	NR	LBBM	Orthostatic hypertension in PD	Orthostatic hypotension causes tachycardia, uncommon falls, disorientation, mental impairment, vision issues, fatigue, and painful shoulders, neck, or low back. They appear when the patient stands up and leave when the patient lies down.	Droxidopa, fludrocortisone, clonidine, transdermal nitroglycerin, nifedipine
9	Cuenca-Bermejo et al. [87] (2021)	NR	LBBM	Cardiac changes in PD	Cardiac anomalies have been observed in PD individuals who do not have sufficient sympathetic innervation in the heart. Hypotension after a meal is followed by supine hypertension; rising blood pressure variability, decreased heart rate and blood pressure, and chronotropic incompetence is all indications.	NR
10	Vikdahl et al. [88] (2015)	147	LBBM	CVD risk in PD	Exercise may be beneficial in lowering the risk of cardiovascular disease in some people. High levels of blood cholesterol, tobacco smoking, and a high BMI have all been associated with the progression of PD.	NR

* SN: serial number, PS: patient size, ME: method of evaluation, Relation: effect of PD on stroke, NR: not reported, SSR: sympathetic skin response, HRV: heart rate variability, OH: orthostatic hypotension, LB: lab-based, MOBI: monoamine oxidase B inhibitors, COMT: catechol-O-methyl transferase inhibitors.

**Table 4:** Pretrained models for COVID-19.

SN	Authors and Citations	Total CT Scan Samples		Pretrained Model	Accuracy (%)
		Positive COVID-19	Negative COVID-19		
1	Halder et al. [206] (2021)	1252	1229	DenseNet 201	97.00
				ResNet50 V2	96.00
				Mobile Net	95.00
				VGG-16	94.00
2	Kumari et al. [189] (2020)	987	921	VGG-16	87.68
				3-layer CNN	56.16
3	Mishra et al. [211] (2021)	360	397	Deep CNN	86.00
4	Saood et al. [175] (2021)	287	314	SegNet	95.00
				Unet	92.00

**Table 7:** Benchmarking scheme for selected studies.

SN	S0	COVID-19 Symptoms in PD Patients						PD Motor Symptoms		PD Non-Motor Symptoms				*Risk Factors*			Gold Standard
		S1	S2	S3	S4	S5	S6	S7	S8	S9	S10	S11	S12	S13	S14	S15	S16
1	Antonini et al. [56] (2020)	✓	✕	✓	✓	✓	✕	✓	✓	✓	✓	✓	✓	✓	✓	✓	PD + COVID-19 + Pneumonia
2	Baschi et al. [7] (2020)	✓	✓	✓	✓	✓	✕	✓	✓	✓	✓	✓	✕	✓	✓	✓	PD + COVID-19 + Respiratory dysfunctions
3	Brown et al. [163] (2020)	✓	✓	✓	✓	✕	✓	✓	✓	✓	✓	✓	✕	✓	✓	✓	PD + COVID-19 + Pneumonia
4	Cella et al. [2] (2020)	✓	✓	✓	✕	✓	✓	✓	✓	✓	✓	✕	✓	✓	✓	✓	PD + COVID-19 + Respiratory dysfunctions
5	Starmbi et al. [129] (2021)	✓	✕	✓	✕	✓	✕	✓	✕	✓	✕	✓	✕	✓	✓	✓	PD + COVID-19 + Respiratory dysfunctions
6	Helmich et al. [6] (2020)	✕	✕	✓	✕	✓	✕	✓	✕	✓	✓	✓	✕	✓	✓	✕	PD + COVID-19 + Pneumonia
7	Khoshnood et al. [5] (2021)	✕	✕	✓	✕	✕	✕	✓	✓	✓	✕	✓	✕	✓	✓	✓	PD + COVID-19 + Respiratory dysfunctions
8	Lau et al. [16] (2021)	✓	✕	✓	✓	✓	✓	✕	✓	✓	✓	✓	✕	✓	✕	✓	PD + COVID-19 + Pneumonia
9	Sulzer et al. [4] (2021)	✓	✓	✓	✕	✕	✓	✕	✓	✕	✓	✓	✕	✓	✓	✓	PD + COVID-19 + Respiratory dysfunctions
10	Tsivgoulis et al. [131] (2021)	✓	✓	✓	✓	✓	✕	✕	✕	✕	✓	✕	✕	✕	✓	✓	PD + COVID-19 + Respiratory dysfunctions
11	Sorbera et al. [130] (2021)	✓	✓	✓	✓	✓	✕	✓	✓	✓	✓	✓	✕	✓	✓	✓	PD + COVID-19 + Pneumonia

S0: Author, S1: Fever, S2: Dry cough, S3: Cough, S4: Shortness of breath, S5: Pneumonia, S6: Delirium, S7: Bradykinesia, S8: Rigidity in throat muscles, S9: Anxiety, S10: Sleep disorder, S11: Hypertension, S12: Fainting, S13: Age, S14: PD duration, S15: PD with COVID-19 and comorbidities, S16: PD with COVID-19 mortality risk factor, ✓: Yes, ✕: No.

**Figure A1:**
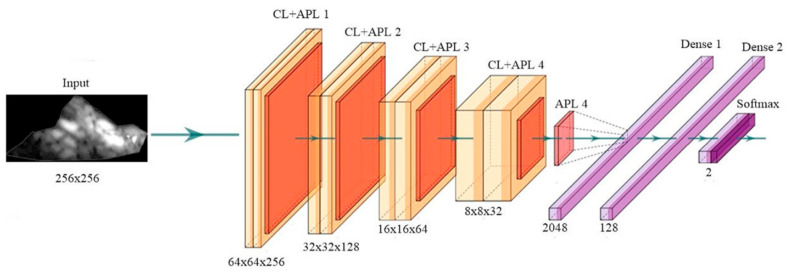
The general structure of CNN architecture (courtesy of AtheroPoint™, Roseville, CA, USA) [292].

**Figure A2:**
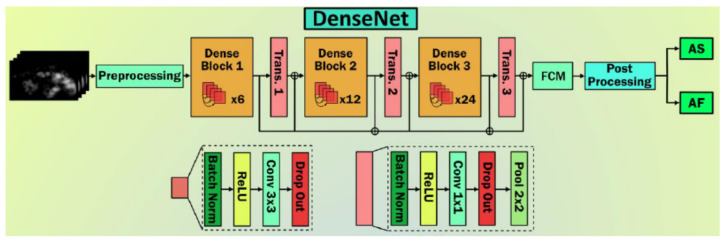
The general structure of Densenet architecture (courtesy of AtheroPoint™, Roseville, CA, USA) [292].

**Figure A3:**
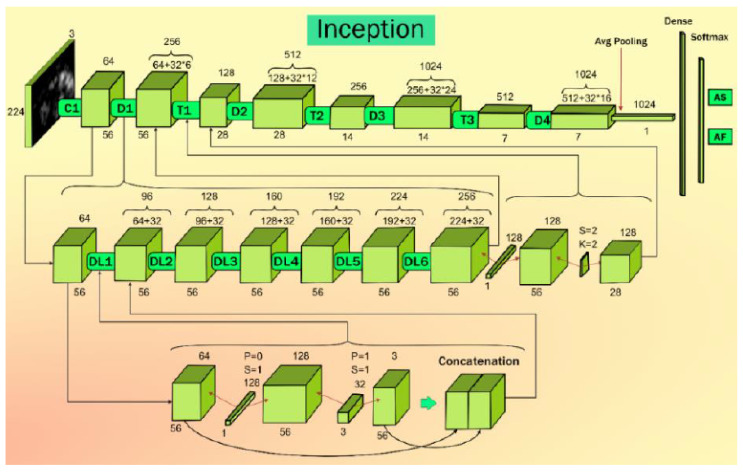
The general structure of InceptionV3 architecture [292].

**Figure A4:**
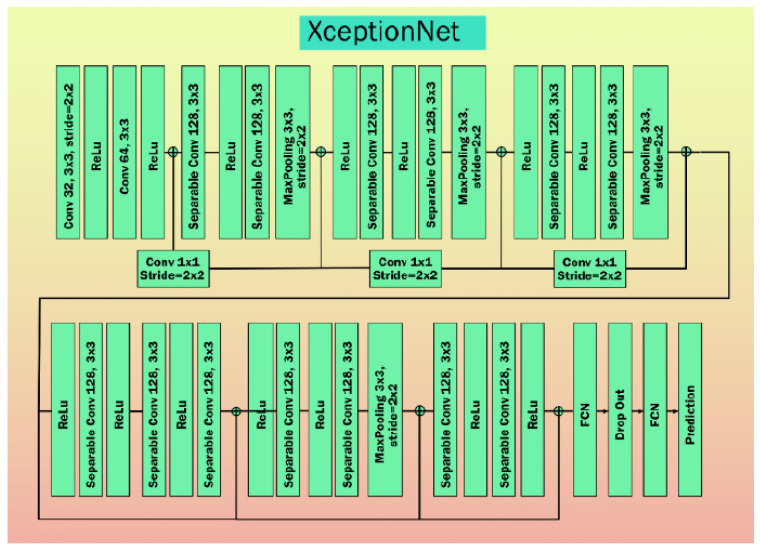
The general structure of XceptionNet architecture [292].

**Figure A5:**
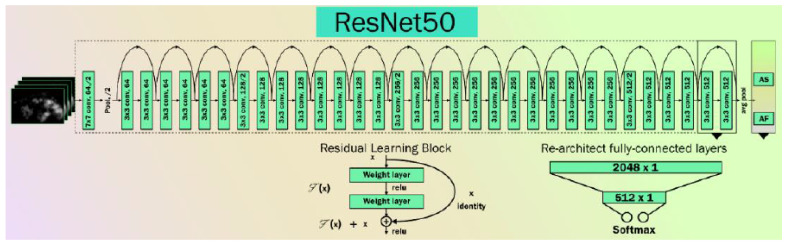
The general structure of Resnet50 architecture [292].

**Figure A6:**
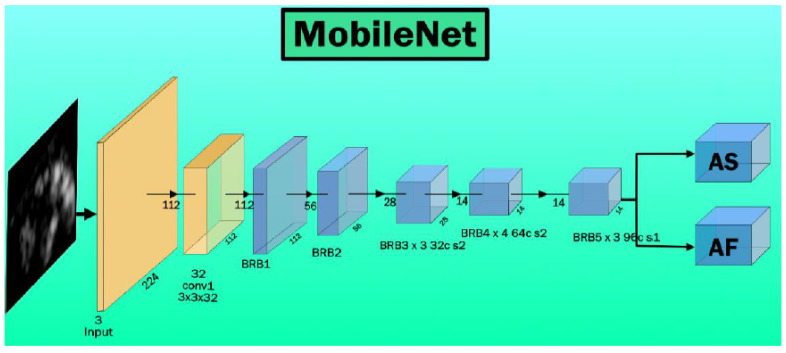
MobileNet architecture, BRB: bottleneck and residual blocks [292].

**Figure A7:**
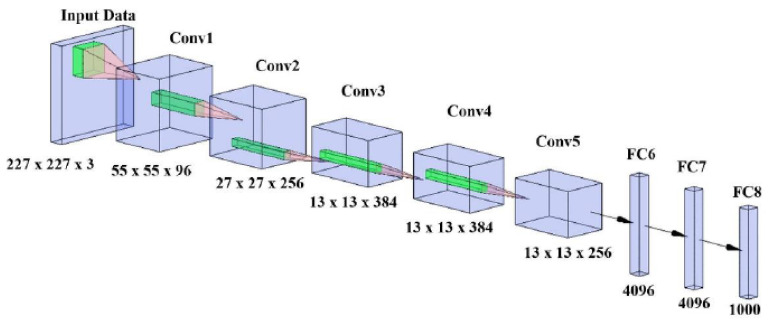
AlexNet architecture [292].

**Figure A8:**
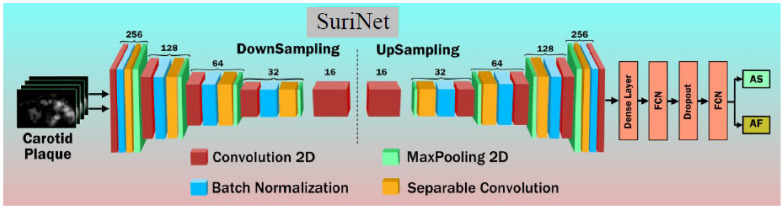
SuriNet architecture [292].

## Data Availability

No data availability.

## Abbreviations

**SN**	**Abb**	**Definition**	**SN**	**Abb**	**Definition**
1	A1c	Glycated hemoglobin	34	VCAM	Vascular cell adhesion molecule
2	ANS	Autonomic nervous system	35	LBBM	Laboratory-based biomarker
3	ANN	Artificial neural network	36	LDL	Low-density lipoprotein
4	ACE2	Angiotensin converting enzyme 2	37	LSTM	Long short-term memory
5	AUC	Area under the curve	38	MedUSE	Medication use
6	AI	Artificial intelligence	39	ML	Machine learning
7	ARDS	Acute respiratory distress syndrome	40	MRI	Magnetic resonance imaging
8	BMI	Body mass index	41	NPV	Negative predictive value
9	CAD	Coronary artery disease	42	NB	Naive byes
10	CAS	Coronary artery syndrome	43	nOH	Neurogenic orthostatic hypotension
11	CCA	circumflex coronary artery	44	Non-ML	Non-machine learning
12	CPD	Chorionic pulmonary disease	45	NN	Neural networks
13	COPD	Chronic obstructive pulmonary disease	46	NR	Not reported
14	CKD	Chronic kidney disease	47	NO	Nitric oxide
15	CT	Computed tomography	48	OBBM	Office-based biomarker
16	CUSIP	Carotid ultrasound image phenotype	49	OH	Orthostatic hypotension
17	CV	Cross-validation	50	PD	Parkinson’s disease
18	CVD	Cardiovascular disease	51	PE	Performance evaluation matrices
19	CNN	Convolution neural network	52	PPV	Positive predictive value
20	DA	Endogenous dopamine	53	PCA	Principal component analysis
21	DL	Deep learning	54	RA	Rheumatoid arthritis
22	DM	Diabetes mellitus	55	RF	Random forest
23	DBP	Diastolic blood pressure	56	RoB	Risk of bias
24	DT	Decision tree	57	RoS	Reactive oxygen species
25	EMG	Electromyography	58	ROC	Receiver operating characteristics
26	EPB	Increased blood pressure	59	RNN	Recurrent neural network
27	FoG	Freezing of gait	60	SCORE	Systematic coronary risk evaluation
28	GGO	Ground-glass opacities	61	SBP	Spontaneous bacterial peritonitis
29	GT	Ground truth	62	RNA	Ribonucleic acid
30	HTN	Hypertension	63	SMOTE	Synthetic minority over-sampling technique
31	HDL	Hybrid deep learning	64	SVM	Support vector machine
32	ICU	Intensive care unit	65	TMD	Temporomandibular disorder
33	ICAM	Intercellular adhesion molecule	66	TMJ	Temporomandibular joint
			67	US	Ultrasound

## Appendix A

### Appendix A.1. Deep Convolutional Neural Network Architecture

Figure A1 shows DCNN’s global architecture. It has four convolution layers and an average pooling layer. The 2D feature map is then flattened to create a 1D map. Two 128-node hidden layers follow. The “Softmax” layer has two nodes representing symptomatic and asymptomatic classes.

### Appendix A.2. DenseNet Architecture

DenseNet architecture solves deep neural nets vanishing gradient problems. This model included dense blocks. It has a pool of convolution layers with 3 × 3 filters to 1 × 1 filters followed by batch normalization (BN) and every layer employs “ReLU” activation. These dense blocks were concatenated using transition blocks. Each transition block includes 2 × 2 to 1 × 1 filters with dropout layers, convolution, and pooling layers. The beauty of DenseNet is that there are parallel connections to avoid losing the features.

### Appendix A.3. Inception V3 Architecture

This model was created to reduce calculation time and parameters. This model handles large datasets. This model is efficient. When using ImageNet, Inception V3 achieves higher accuracy. The model has blocks. Convolution and max-pooling layers are included. Figure A3 shows architecture, DL1 to DL6 as depth-wise convolution, C1 as initial convolution, T1 to T3 as transition layer, and D1 to D4 as batch normalization. In Inception V3, each top-row block represents rows 2 and 3. Row 2 repeats row 3. Each convolution layer has a stride 1 and padding 0 convolution filter. First, a 3 × 3 convolution layer with stride 1 and padding 1 is added to the feature map (FM). It reduces FM depth; the resultant FM and initial FM are fused to make row 2 blocks.

### Appendix A.4. Xception Net Architecture

A modification to IV3 was suggested by Chollet et al. from Google. This modification would involve replacing the inception modules with modified depth-wise separable convolution layers. There are a total of 36 strata in this architecture. XceptionNet is lightweight compared to IV3, and it contains the same number of parameters as IV3. The top-1 accuracy of this architecture is 0.790, and the top-5 accuracy is 0.945, which is better than InceptinV3’s performance. The Xception system’s architecture is depicted in Figure A4.

### Appendix A.5. Resnet50 Architecture

To solve the vanishing gradient problem the ResNet model is used. Skip connections are in residual blocks. These skip connections skip training levels and output links. Skipping layers allows the model to learn complex patterns. This TL model uses CIFAR-10 data, unlike others. Figure A5 depicts ResNet. Architecture pairs two 3 × 3 convolution layers. These pairs’ outputs and inputs are combined and fed to the following pair. Here, 64–512 filters are listed. After the last 3 × 3 convolution layer with 512 filters and a flattened layer for vectorizing 2D features, the output is predicted using softmax activation.

### Appendix A.6. MobileNet Architecture

This was the first computer vision model developed for TensorFlow for mobile devices. It contains 28 layers and uses the TFlite (database) library. Figure A6 presents the architecture of MobileNet architecture. This model contains bottleneck residual blocks (BRBs), also referred to as inverted residual blocks used for reducing the number of training parameters in the model.

### Appendix A.7. AlexNet Architecture

Alex Krizhevsky et al. suggested AlexNet for ImageNet in 2012. It is CNN’s initial architecture for computer vision challenges. This architecture’s error rate is 15.3%. This architecture transforms AI. It accepts 256 256 images and has five convolution layers and two completely connected networks. Softmax output layer. Sample architecture is shown in Figure A7.

### Appendix A.8. Suri Net Architecture

The first is a more traditional CNN, and the second is an architecture known as SuriNet. Although the UNet network is often used for segmentation in medical image analysis, the SuriNet architecture, which is a modified version of the UNet architecture, was the one that we relied on for classification. We employed separable convolution neural networks in the SuriNet architecture that we suggested to cut down on the amount of overfitting and the number of parameters that were necessary for training. The architecture of SuriNet is depicted in Figure A8.
